# Compaction of chromatin domains regulates target search times of proteins

**DOI:** 10.1371/journal.pcbi.1013843

**Published:** 2026-01-20

**Authors:** Shuvadip Dutta, Adarshkrishnan Rajakumar, Ranjith Padinhateeri, Mithun K. Mitra

**Affiliations:** 1 Department of Physics, Indian Institute of Technology Bombay, Mumbai, Maharashtra, India; 2 School of Mathematical Sciences, Queensland University of Technology (QUT), Brisbane, Queensland, Australia; 3 ARC Centre of Excellence for the Mathematical Analysis of Cellular Systems, QUT, Brisbane, Queensland, Australia; 4 Department of Biosciences and Bioengineering, Indian Institute of Technology Bombay, Mumbai, Maharashtra, India; 5 Sunita Sanghi Centre of Aging and Neurodegenerative Diseases, Indian Institute of Technology Bombay, Mumbai, Maharashtra, India; 6 Koita Centre for Digital Health, Indian Institute of Technology Bombay, Mumbai, Maharashtra, India; LJI: La Jolla Institute for Immunology, UNITED STATES OF AMERICA

## Abstract

Protein molecules must efficiently locate specific DNA sequences within the densely packed chromatin of the cell nucleus. We investigate how the spatial organisation of chromatin, specifically its organisation into Topologically Associating Domains (TADs), fundamentally affects this search process. Using exact analytical theory and simulations of different models of chromatin, we show that target search within compact, highly connected chromatin domains can leverage intersegmental jumps to significantly decrease search times. Further, we establish that there exists an optimal degree of polymer compaction that minimizes the search time for proteins to find their targets. For highly folded domains, our results suggest that rather than bulk diffusion, intersegmental transfers – jumping between chromatin segments that are close together in space – drive the optimal search process. Remarkably, when we analyse 8,355 TAD structures across the human genome, we find that their natural connectivity matches with the theoretical optimum predicted by our model. The structural organisation within TADs significantly reduces protein search times far beyond what is achievable through classical facilitated diffusion. In essence, our work suggests that packaging of chromatin inside the nucleus has implications beyond spatial organisation, and is also intricately linked to the dynamics of proteins inside the nuclear environment.

## Author summary

Inside the dynamic world of cells, proteins must quickly locate specific sequences on chromatin to bind and execute their functions. In this manuscript, we explore how proteins find their targets within densely packed chromatin—the combination of DNA and proteins that form chromosomes. Using exact analytic theory and computer simulations, we studied how the three-dimensional organisation of chromatin—especially into structures called Topologically Associating Domains (TADs)—affects the ability of proteins to locate their targets. We show that the topology of chromatin helps proteins search for their targets by jumping between spatially adjacent chromatin segments. Remarkably, we show that chromatin has an optimal connectivity which minimises the search time to locate the target. We then analysed experimental data for thousands of TADs from human chromosomes to show that the natural organisation of TADs falls within this optimum region that minimises search times. This suggests that chromatin topology plays a crucial role not only in spatial organisation, but also enables faster protein search and hence aids gene regulation.

## 1 Introduction

The mechanism of target search by DNA-associated proteins such as Transcription Factors (TFs) inside the nucleus remains an open question [1–7]. The leading proposed candidate is *facilitated diffusion* [4,8,9]—where the protein searches for its target through repeated rounds of one-dimensional non-specific sliding along DNA and three-dimensional diffusion in bulk. However, while some studies have investigated experimental evidence for the facilitated diffusion mechanism in prokaryotes [10,11], evidence in eukaryotic organisms remains scant [12]. To explore the dynamic movements of proteins along DNA strands to locate specific target sites within large genomic regions, four distinct search mechanisms have been proposed: 1D sliding, hopping or jumping, 3D diffusion, and intersegmental transfer [13]. Numerous *in vitro* studies have delved into these mechanisms to shed light on how proteins interact with and navigate along DNA. One-dimensional sliding of proteins like RNA polymerase, Rad51, hOgg1 etc. has been observed using total internal reflection fluorescence (TIRF) microscopy and C-trap optical tweezers [14–17]. Experiments have also revealed an intermediate, partially dissociated state in protein-DNA interactions, where proteins can be captured by a second DNA segment, resembling “direct transfer” reactions [18]. While such intersegmental hops have been long known in the literature, most theoretical studies assume that intersegmental transfer moves the protein to an uncorrelated segment and hence has the same effect as 3D diffusion [2]. Thus most theoretical models use a combination of 1D sliding and bulk 3D diffusion, and the predominant understanding is that a balance between these two processes can lead to an optimisation of search times, compared to a pure 1D or a pure 3D search [8].

In contrast to *in vitro* studies, experimental data on the spatial organisation of chromatin inside nuclei has now established, however, there exists densely packed chromatin regions with high density and viscosity [19], which hinder free diffusion in the solution phase [13]. In densely packed bacterial nucleoids, experiments have highlighted a spectrum of mechanisms for protein mobility, including direct transfer and hopping, leading to generally sluggish protein diffusion within bacterial cells [20]. Moreover, in a more recent single-molecule study, it was demonstrated that within phase-separated condensed chromatin, non-specific multivalent interactions slow the mobility of proteins, limiting their capacity for free 3D diffusion [21]. Thus, the spatial organisation of chromatin within nuclei may play a key role in determining the efficiency of various search strategies. In particular, for chromatin domains with high densities, 3D diffusion may not offer an efficient pathway for proteins to navigate to new segments of the genome. In contrast, intersegmental transfer from genomically distant DNA segments, which preserves spatial correlations over long length scales offers a distinct search mechanism for effective search that does not include free diffusion in the bulk [13,22].

**Fig 1:**
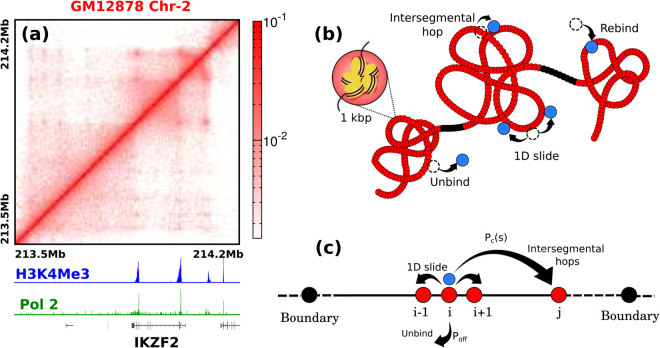
TAD organisation influences chromatin conformation and allowed intersegmental hops. **(a)** A representative contact domain shows TADs as square blocks of increased interaction frequencies [42]. The appearance of a TAD is often accompanied by H3K4Me3 and Pol-2 enrichment peaks at the boundaries, as well as the activation of a gene whose promoter lies at one of the peak loci. **(b)** Coarse-grained (1 kbp) polymer representation of chromatin with compact TAD regions (red) separated by relatively extended boundary regions (black). The blue bead represents a protein. During the search process, the protein can move along the polymer backbone via one-dimensional sliding. It can also unbind from the backbone and rebind to a randomly chosen polymer bead. Additionally, intersegmental hopping allows the protein to jump to a genomically distant polymer bead that is spatially close in three dimensions. **(c)** A contact space representation of TAD-like polymer domains. Each bead is connected via bonds to its neighbour along the backbone. In addition, bonds between genomically distant but spatially neighbouring beads allow for intersegmental jumps. Black beads denote the boundaries of the TAD.

This mechanism assumes particular importance for mammalian cells, where numerous theoretical and experimental studies have delved into the hierarchical organisation of the genome across various length scales [23–29]. A ubiquitous feature of this spatial organisation is that at the scales of up to a few megabases, the genome is organised into compact regions, known as Topologically Associating Domains (TADs) (Fig 1a) [30–32]. TADs are structural and functional units of the genome characterised by large squares of increased contact frequency tiling the contact matrices’ diagonal [31] (Fig 1a). This implies higher interaction frequencies between regions within the same TAD [33]. Concurrent with this high intra-domain contacts inside TADs, is a relative insulation among adjacent domains through TAD boundaries [34,35]. TAD boundaries, marked by the enrichment of H3K4Me3 and the presence of abundant Pol2 [36], serve as hubs where the promoter regions of numerous transcriptional units are strategically located (Fig 1a). The TAD structure is maintained through loop extruding proteins such as cohesins, which bring together non-neighbouring genomic regions to form a looped organisation leading to enhanced contacts [37]. TADs are known to co-localise genes and their regulatory elements and hence this organisation has functional implications in the context of gene expression [38]. At Mbp scales, the contact probability—the probability of two genomic regions separated by *s* base pairs coming into contact—follows a power-law, $P_{c}(s)∼s^{-\gamma }$, with an average γ∼1.08 [39]. However, within TADs, the power-law exponent decreases to less than 1 due to the enhanced frequency of intra-TAD contacts [34,40]. These enhanced contacts result in a higher occurrence of long intersegmental hops (Fig 1b), consequently influencing the kinetics of search processes of proteins on chromatin [41].

Protein diffusion over general polymer chains, with allowed jumps to all Euclidean (but not necessarily chemical) nearby locations, has been studied to characterise transport coefficients [43]. Such a random walk, on frozen polymer chain structures, is surprisingly diffusive. The transport coefficients show a gradual transition to a super-diffusive regime upon introduction of annealing [44]. Thus, a diffusing protein’s search performance can be improved to super-diffusion by incorporating appropriate polymer dynamics and fluctuations. A related quantity in such stochastic processes is the completion time of transport which may lead to important biological events, or search times for specific binding sites, referred to in the literature as first passage times [6]. An estimate of first passage times for proteins diffusing on a polymer backbone characterised by increased long-range interaction frequencies as in TADs has implications for many biological processes [45]. DNA conformations and looped structures such as rosettes have also been predicted to affect search kinetics [46–48].

In this article, we investigate the role of 3D chromatin organisation, in particular within compact TAD-like regions, in the target search process by proteins. We show how the search times of proteins crucially depend on the topology of polymer domains characterised by long-range intersegmental contacts. In biologically relevant regimes, we show that 3D diffusion is not an efficient search strategy compared to search mediated through intersegmental jumps. Finally, we analyse available experimental data to show that conformations of human TADs are optimised for efficient search.

## 2 Model and methods

### 2.1 Contact space representation of polymer topology

We propose a theoretical framework to model a compact polymer domain of length *L* as a network in contact space. In this model, each bead (or node) of the polymer represents a coarse-grained chromatin segment of size 1 kbp. The conformation of the polymer is encoded in a matrix *C*_*ij*_ of dimension (L  +  1) × (L  +  1), where i,j∈[0,L]. Two beads *i* and *j* are assumed to be in contact with a probability $P_{c}(s)=cs^{-\gamma }$, where s=|i−j|>1 and i,j∈[1,L−1]. The polymer topology enforces $C_{i,i+1}=C_{i-1,i}=1$ for all *i*. The exponent *γ* serves as a measure for polymer compaction. Initially, we assume the domain to be a uniform network by setting γ=0, leading to a uniform contact probability $P_{c}(s)=c\equiv p_{u}$. Here, *p*_*u*_ effectively quantifies the compaction of the domain: a low *p*_*u*_ represents a 1D chain, whereas $p_{u}\to 1$ corresponds to a fully connected network. For a broader perspective, we extend the model to networks with power-law contact probabilities for varying values of the contact probability exponent *γ*. As the polymer collapses, contacts become more frequent, resulting in smaller values of *γ*. For a self-avoiding polymer, γ=2.2, which decreases to γ=1.5 for a random walk polymer. Chromosomes, on average, exhibit γ≈1.08 [39], while experiments within highly folded TADs report even smaller *γ* [34]. We construct power-law networks that follow $P_{c}(s)=cs^{-\gamma }$ for different *γ* values, closely mimicking polymer domains with different levels of compaction. Unless stated otherwise, we use *c* = 0.0214, consistent with experimentally observed distributions of *c*. The polymer connectivity depends both on the number of intersegmental contacts, and on their distribution (*γ*), and these together determine the compactness of the polymer domain. In general, for similar numbers of intersegmental contacts, a larger exponent implies longer loops, and hence more compact domains. For both the uniform and power-law networks, we interpret our results in terms of the polymer compaction, although this is achieved only through varying the average number of contacts in the uniform case, and both the number and their distribution for power-law networks.

### 2.2 Protein as random walker

In this network representation, a protein is modelled as an unbiased random walker navigating within a polymer domain (i,j∈[1,L − 1]). At each time step (*τ*), a protein located at bead *i* can either slide along the chain to one of its 1D neighbours (*i*  +  1 and *i*–1) or hop to a genomically distant (but spatially adjacent in 3D) bead *j* if *C*_*ij*_ = 1 (Fig 1b, 1c). Additionally, proteins can unbind from the polymer with a probability $p_{off}$, entering the bulk phase. While in the bulk, the protein explores the solution phase with a mean exploration time of $\tau_{f}=100\tau$ [49] before reattaching to the polymer. We assume the exploration time is large enough that the memory of the unbinding site is lost, and the protein rebinds randomly to any site of the polymer, consistent with previous studies [49,50]. When the protein reaches either boundary site, it is considered to have successfully reached the target.

### 2.3 Static and dynamic polymer domains

We consider two scenarios based on whether the connectivity matrix evolves during the target search process. If the polymer configuration (*C*_*ij*_) remains constant over time, the target search occurs on a static polymer configuration. However, chromatin organisation within a cell is now widely recognised as a dynamic structure rather than a static one [51]. This dynamic behaviour arises from various factors, including tethering of chromatin to the nuclear lamina, interactions with diverse proteins, and the dynamic process of loop extrusion mediated by cohesin [52]. If *C*_*ij*_ evolves with time, the target search takes place on a dynamic polymer. Below, we outline the procedure for generating the connectivity matrix and the simulation methodology for both scenarios.

#### 2.3.1 Generation of the connectivity matrix.

Given a contact probability *P*_*c*_(*s*) we construct a connectivity matrix *C*_*ij*_. To construct this matrix, we choose a random pair of monomers *i* and *j*, and set *C*_*ij*_ = 1 with probability *p*_*u*_ for the uniform network case, and with probability $c|i-j{|}^{-\gamma }$ for the power-law networks. We repeat this procedure for all pairs (except nearest neighbours) to construct the full matrix. For nearest neighbours, by construction, *C*_*i*,*i* + 1_ = 1.

#### 2.3.2 Search time on static networks.

To determine the mean search time ($\left\langle T_{search}\right\rangle$) within the constructed polymer domain for static networks, the protein is initially positioned at the midpoint of the domain ($i=\frac{L}{2}$). The time taken for the walker to reach either of the absorbing boundaries (i=0,L) for the first time is recorded. The mean search time ($\left\langle T_{search}\right\rangle$) is then calculated by averaging the recorded times over an ensemble of realisations, encompassing different configurations of *C*_*ij*_ and random walker trajectories.

#### 2.3.3 Search time on dynamic networks.

We also investigate how search times change when the polymer conformation is not fixed, but changes randomly during the search process. This involves reshuffling the positions of non-neighbouring bonds (*C*_*ij*_ = 1) at regular time intervals, denoted as $t_{config-switch}$. When $t_{config-switch}=\tau$, we call the configuration fully dynamic as it changes with each step of the protein. Conversely, when $t_{config-switch}>>\tau$, the configuration is effectively static. Note that when the configuration of the underlying network is updated, it retains no memory of the previous configuration, effectively implying that polymer relaxation times are smaller than $t_{config-switch}$. The mean search time ($\left\langle T_{search}\right\rangle$) is then calculated by averaging the recorded times over different trajectories.

### 2.4 Theoretical calculation of search times

We compute the configuration-averaged mean search times $\left\langle T_{search}\right\rangle$, defined as the time a protein takes to reach either boundaries of the polymer for the first time. The mean search time can be computed as


$$𝕋={𝔸}^{-1}𝔹$$

where ${𝕋}_{i}$ is the time taken to reach the target starting from the *i*^*th*^ site. The matrices 𝔸 and 𝔹 are defined by,

$${𝔸}_{ij}=\delta_{i,j}-\frac{1-p_{off}}{N_{i}}C_{ij}-p_{off}\delta_{j,f},\;\forall i\neq 0,L,f,\;{𝔸}_{fj}=\delta_{f,j}-\frac{1-\delta_{f,j}}{L+1},\;{𝔸}_{ij}=\delta_{i,j},\;\text{for }i=0,L,\;{𝔹}_{i}=\tau +(\tau_{f}-\tau )\delta_{i,f}-\tau (\delta_{i,0}+\delta_{i,L})$$ (1)

where, *τ* is the time taken for a single step, *C*_*ij*_ denotes the connectivity matrix, and *N*_*i*_ denotes the total number of bonds for the *i*^*th*^ site. The site *i* = *f* denotes the freely diffusing state in solution. For a single configuration, the variable *C*_*ij*_ takes the value 1 with probability *P*_*c*_(*s* = |*i*−*j*|), given by

$$P_{c}(|i-j|)=\left\{ \begin{array}{cc} \delta_{|i-j|,1}+(1-\delta_{|i-j|,1})p_{u} & uniform\\ \delta_{|i-j|,1}+(1-\delta_{|i-j|,1})c|i-j{|}^{-\gamma } & power\;law \end{array}\right.$$ (2)

Eqs 1 and 2 can be solved to obtain estimates of the search time to find the target.

### 2.5 Polymer models of chromatin

In order to validate our results obtained from the contact space representation, we simulate search on topologies obtained from three polymer models - the freely rotating chain, the Lennard-Jones bead spring polymer, and a soft Lennard-Jones polymer.

#### 2.5.1 Freely rotating chain model.

We simulate the freely rotating chain (FRC) model which has been extensively used to investigate chromatin behaviour [53,54]. In this model, the orientation of neighbouring bonds is restricted to lie within a cone that subtends an angle *θ* [55]. The angle *θ* regulates the polymer’s compaction, with larger values of *θ* leading to a collapsed state.

#### 2.5.2 LJ bead-spring polymer model.

We simulate a bead-spring polymer consisting of *L* beads each of size (diameter) *σ*. The total energy (*E*) of the polymer is given by-


$$E=\sum_{i=1}^{L-1}E_{spring}(|{\vec{r}}_{i+1}-{\vec{r}}_{i}|)+\sum_{i=1}^{L-1}\sum_{j=i+1}^{L}E_{LJ}(|{\vec{r}}_{j}-{\vec{r}}_{i}|)$$

Here ${\vec{r}}_{i}$ is the position vector of *i*^*th*^ bead. The first term corresponds to the energy contribution from harmonic springs ($E_{spring}(r)=\frac{k}{2}(r$ − $\sigma {)}^{2}$) that connect adjacent beads along the chain. The second term describes attractive interactions governed by the Lennard-Jones (LJ) potential:


$$E_{LJ}(r)=\left\{ \begin{array}{cc} 4\epsilon \left[{\left(\frac{\sigma }{r}\right)}^{12}-{\left(\frac{\sigma }{r}\right)}^{6}\right] & r<2.5\sigma \\ 0 & r\geq 2.5\sigma \end{array}\right.$$

Here, *ϵ* denotes the strength of the attractive interaction.

#### 2.5.3 Soft LJ bead-spring polymer model.

For the soft LJ polymer, the energy (*E*_*soft*_) is given by [28]:


$$E_{soft}(r)=\left\{ \begin{array}{cc} V_{0}[1-(\frac{r}{r_{m}}{)}^{\eta_{1}}{]}^{\eta_{2}}-\epsilon & 0\leq r<r_{m}\\ 4\epsilon \left[{\left(\frac{\sigma }{r}\right)}^{12}-{\left(\frac{\sigma }{r}\right)}^{6}\right] & r_{m}\leq r<2.5\sigma \\ 0 & r\geq 2.5\sigma \end{array}\right.$$

The expression *V*_0_ − *ϵ* denotes the energy penalty associated with complete overlap, and the parameters $\eta_{1}$ and $\eta_{2}$ are utilised to adjust the softness of the potential, as illustrated in S12 Fig. For *r* < *r*_*m*_, the potential is repulsive but softer than the standard LJ potential. For $r_{m}\leq r<2.5\sigma$, the potential transitions to the LJ form, inducing attractive interactions. Beyond the cut-off distance (r≥2.5σ), the interaction energy is set to zero. The parameters used in this study are: $V_{0}=10^{7}$, $\eta_{1}=$ variable, $\eta_{2}=3.16$, *r*_*m*_ = 1.12, σ=1.

#### Simulation of bead-spring polymer.

The Langevin dynamics simulations were performed using the LAMMPS software package [56]. In this approach, the position of each polymer bead evolves according to the equation:


$$m_{i}\frac{d^{2}r_{i}}{dt^{2}}=-\nabla E_{i}-\gamma_{i}\frac{dr_{i}}{dt}+\sqrt{2\gamma_{i}k_{B}T}\eta_{i}(t)$$

where *r*_*i*_ is the position of the center of mass of the *i*-th bead, *m*_*i*_ is its mass, *E*_*i*_ is the sum of all the interaction potentials acting on bead *i*. The friction coefficient $\gamma_{i}$ determines the diffusion constant $D_{i}=k_{B}T/\gamma_{i}$. For polymer beads, we set *m*_*i*_ = 1 and $\gamma_{i}=1.0$. $\eta_{i}(t)$ is a noise term with components which satisfy: $\langle \eta_{i\alpha }(t)\rangle =0,\;\langle \eta_{i\alpha }(t)\eta_{j\beta }(t^{\prime })\rangle =\delta_{ij}\delta_{\alpha \beta }\delta (t$ − $t^{\prime })$, where $\eta_{i\alpha }(t)$ is component *α* of the noise vector for object *i*, and $\delta_{ij}$ and δ(t) are the Kronecker and Dirac delta functions respectively. These equations are solved using a velocity-Verlet algorithm with time step dt=0.01τ, where *τ* is the simulation time unit defined by $\tau =(m\sigma^{2}/k_{B}T{)}^{\frac{1}{2}}$.

#### 2.5.4 Simulation of protein walk on polymer.

We transform simulated polymer structures with varying compaction levels into network representations (*C*_*ij*_). This transformation is achieved by defining a cut-off radius (*r*_*c*_), which determines contact between non-bonded beads. Specifically, if the 3D distance (*r*_*ij*_) between two non-bonded beads (*i*,*j*) in the polymer structure is less than or equal to the cut-off radius, they are considered in contact, and *C*_*ij*_ is set to 1. Otherwise, *C*_*ij*_ is set to 0:


$$C_{ij}=\left\{ \begin{array}{cc} 1 & r_{ij}\leq r_{c}\\ 0 & r_{ij}>r_{c} \end{array}\right.$$

We also calculate total number of non-neighbouring bonds (*N*_*b*_) for a given configuration, which is defined as,


$$N_{b}=\sum_{j=i+2}^{L}\sum_{i=1}^{L}C_{ij}$$

Using these network representations, we perform random walks and measure the time required to reach target. The results are averaged over 10,000 independent runs each across 1,000 different configurations to compute $\left\langle T_{search}\right\rangle$.

### 2.6 Construction of TADs from experimental Hi-C data

To study the protein search process within realistic chromatin architecture, we used chromatin conformation capture data from Hi-C experiments. Our analysis was based on a high-resolution 1 kb Hi-C map derived from the GM-12878 human lymphoblastoid cell line [42]. Specifically, we utilised KR-normalised [57] Hi-C contact frequency matrices *f*_*ij*_ for all autosomes (Chr1–Chr22). We then determine the maximum value of the non-diagonal (i≠j) contact frequency for each chromosome and divide each *f*_*ij*_ by this number to derive a contact probability map (*P*_*ij*_) where all elements are scaled between 0 and 1 [58]. Furthermore, our analysis of topologically associating domains (TADs) relied on a curated set of 8,355 TADs from [42], annotated using the Arrowhead algorithm.

**Method-1:** We define intra-TAD contact probability *P*_*c*_(*s*) of two points separated by genomic distance *s* as the average contact probability between of all genomic loci separated by genomic distance *s* within a TAD. Contact probability exhibited a power-law, within a range of values of *s*. We measured *γ* as the slope of the best-fit line and *c* as the *y*-intercept, when plotted on log-log axes, within a chosen range of distances. The obtained *γ* value signifies the compaction of the corresponding TAD. We calculate the average search time ($\left\langle T_{search}\right\rangle$) for each TAD using our theoretical framework with *c* and *γ* as input.

**Method-2:** To simulate the dynamics of a protein within a realistic chromatin structure, we employ a previously described model based on sliding and intersegmental jumping on a network. This network replicates the chromatin architecture and interactions within a single TAD in 3D space. The network is constructed using *P*_*ij*_ values derived from experimental Hi-C data as follows:


$$C_{ij}=\left\{ \begin{array}{cc} 1 & \text{if }r_{n}\leq P_{ij},\\ 0 & \text{if }r_{n}>P_{ij}, \end{array}\right.$$

where *r*_*n*_ is a uniform random number in the range [0,1]. Here, *C*_*ij*_ represents the connectivity of the chromatin polymer within a TAD. In addition to the connectivity derived from *P*_*ij*_ using Hi-C data, we enforce $C_{i,i+1}=C_{i,i-1}=1$ to maintain polymer connectivity along the backbone. After constructing the network based on Hi-C data, we simulate protein dynamics within this chromatin network. The ensemble average search time ($\left\langle T_{search}\right\rangle$) is calculated by averaging over 1,000 distinct configurations, with 10,000 search simulated in each configuration.

## 3 Results

### 3.1 Search time varies non-monotonically with compaction of domain

**Fig 2:**
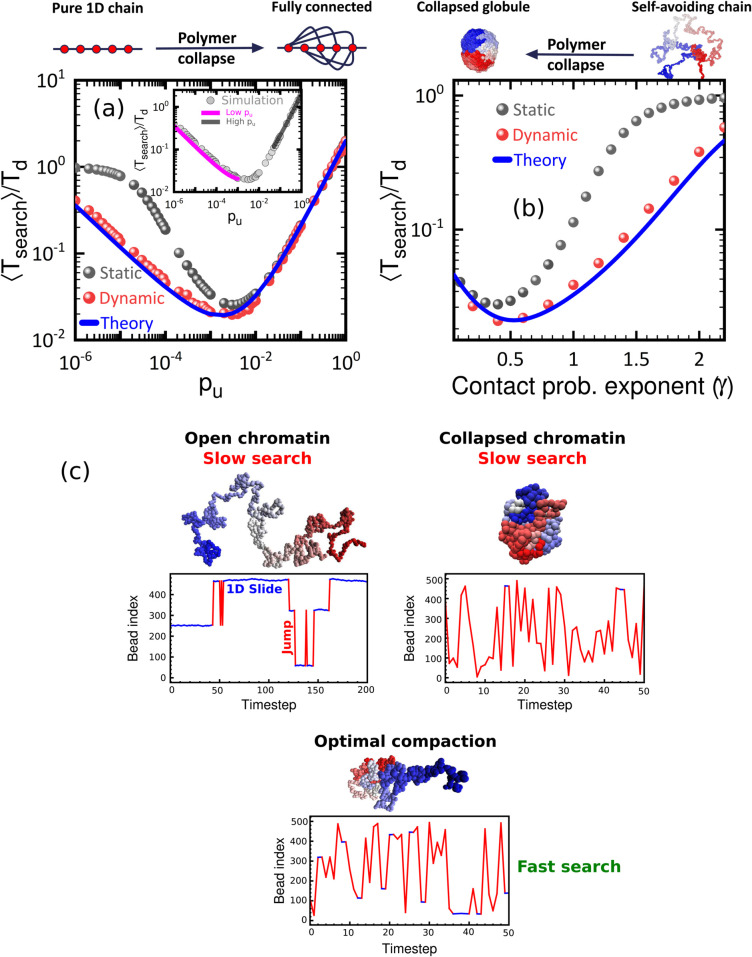
Search times with 1D sliding and intersegmental hops are minimised for an optimal polymer compaction. **(a)** Scaled mean search time $\left(\frac{\left\langle T_{search}\right\rangle }{T_{d}}\right)$ for static (black) and dynamic (red) conformations in a uniformly connected network domain vs probability of contact (*p*_*u*_) between any two non-neighbouring beads for *L* = 500. The solid blue line is the exact analytical solution of Eqs 1 and 2 (see also S2 Text). The inset shows the low-*p*_*u*_ and high-*p*_*u*_ approximations from Eqs 3 and 4. **(b)** Scaled mean search time $\left(\frac{\left\langle T_{search}\right\rangle }{T_{d}}\right)$ in power-law connected network domain as a function of contact probability exponent(*γ*) for *L* = 500 also shows non-monotonic behaviour for both static (black) and dynamic (red) configurations. The blue line is the theoretical calculation from Eqs 1 and 2. **(c)** For open conformations (low *p*_*u*_ or, high *γ*), the search is dominated by 1D sliding (blue segments), with very few intersegmental hops, resulting in slow target search. For collapsed conformations (high *p*_*u*_ or, low *γ*), due to many intersegmental hops (red segments), search times are high. At optimal compactions, balanced 1D sliding and intersegmental hops leads to an optimisation of search times.

Motivated by experimental results of very high estimates of time a protein spends non-specifically bound to the DNA [11,59–62], and consistent with high volume fractions inside TADs (S1 Text, S1 Fig) [63], we first consider the limiting case $p_{off}=0$ (see Sect 2), so that target search happens only along the polymer, via 1D sliding and intersegmental jumps. For simplicity, we first assume γ=0, so that all beads have a uniform connection probability, $P_{c}(s)=c\equiv p_{u}$. We observe a non-monotonic relation between $\left\langle T_{search}\right\rangle$ and *p*_*u*_ (Fig 2a). When *p*_*u*_ = 0, the protein performs a 1D random walk, giving $\langle T_{search}\rangle =\frac{L^{2}}{8D}\equiv T_{d}$ where $D=\frac{1}{2\tau }$ [64]. As *p*_*u*_ increases, more paths to reach the boundaries emerge, reducing $\left\langle T_{search}\right\rangle$. However, beyond a threshold connectivity, $\left\langle T_{search}\right\rangle$ increases due to excess intersegmental bonds diverting the walker. This non-monotonic behaviour of $\left\langle T_{search}\right\rangle$ is independent of whether the polymer network is static (Fig 2a black symbols) or dynamic (Fig 2a red symbols, and S2 Figa). For dynamic polymer networks, we obtain an *exact* analytic expression for the search time as a function of the connection probability (See S2 Text). Dynamic rewiring of network connections reduces $\left\langle T_{search}\right\rangle$, with faster configuration changes yielding lower $\left\langle T_{search}\right\rangle$ at low *p*_*u*_ (S2 Figa). However, at high *p*_*u*_ values, network dynamicity offers little advantage, as most nodes already have numerous long-range connections. Systematically increasing the switching time relative to the protein sliding timescale allows us to smoothly interpolate between the dynamic and static results (S2 Figa). At low *p*_*u*_, to leading order, we have,

$$\frac{\left\langle T_{search}\right\rangle }{T_{d}}\overset{p_{u}\to 0}{\approx }1-\frac{\left(L^{3}-5L^{2}-20L+48\right)}{48}p_{u}\;for\;p_{u}\ll \frac{1}{L^{2}}$$ (3)

a decreasing function of *p*_*u*_ (see S2 Text). At high connection probability, as $p_{u}\to 1$, all sites become equivalent, and a simplified governing equation can be solved to yield (see S2 Text),

$$\langle T_{search}\rangle \overset{p_{u}\to 1}{=}2+(L-3)p_{u}+\frac{(2+(L-4)p_{u}{)}^{2}}{2p_{u}}$$ (4)

which implies a linear increase with *p*_*u*_ as $p_{u}\to 1$, also consistent with a solution using the effective medium approach [65] (see S2 Text, S5 Fig). As can be seen from Fig 2a inset, the analytic expression at both small and high *p*_*u*_ limits matches the simulation results. The optimal behaviour of $\left\langle T_{search}\right\rangle$ is consistent for differential rates of sliding and intersegmental jumps (S2 Figb), for all polymer lengths (S3 Fig) and different initial protein positions (S4 Fig). Also, while we have assumed that the target site at the boundary is insulated from the interior sites motivated by earlier studies [32,66], our central result is independent of this strong insulation, and persists when the protein can hop directly to the target site with low (but finite) rates (S6 Fig). Note that in the two extreme limits, this uniform network construction does not represent valid polymer conformations. As $p_{u}\to 0$, there are no intersegmental bonds, implying a pure rod-like conformation. In the opposite limit $p_{u}\to 1$, all monomers are connected to all other monomers, which is not possible for physical polymers. Hence the search times in these limits may not be physical. Further, for real polymers, the contact probability scales with the genomic separation. Despite these assumptions, this simple analytically tractable model allows to capture the non-monotonic behaviour of search times with increasing compaction, and hence, intersegmental jumps.

Having established this central result in the case γ=0, we now turn to the more realistic scenario in which we introduce intersegmental hops with a power-law connectivity, γ≠0 (see Sect 2). For a self-avoiding polymer (SAW), γ=2.2, which reduces to γ=1.5 for a random walk (RW). For chromosomes, on average, γ≈1.08 [39], while within highly folded TADs, experiments report even smaller values for *γ* [40]. Analysis of Hi-C data inside single TADs have reported contact probability exponents as low as γ=0.14 [67]. As a polymer undergoes collapse, contacts become more frequent, reducing the contact probability exponent *γ*. We explore the behaviour of search times across the full biologically and physically relevant range of 0.1≤γ≤2.2. We observe a non-monotonic behaviour of the search times for both and static and dynamic realisations of such power-law networks, as shown in Fig 2b. Again, when the polymer is relatively open (larger *γ*), dynamic rewiring of the connectivities helps reduce search times, similar to the observation from the simpler uniform connectivity ($P_{c}(s)=p_{u}$) case. As the polymer compacts from a self-avoiding conformation, search times can decrease by an order of magnitude, followed by a rise of up to five times at very low *γ* compared to the minimum search time. Note that the minimum *γ* occurs in the regime γ<1, suggesting a possible connection with observed compaction in folded TAD domains. The precise location of the optimum *γ* value depends on the choice of *c*. We verify that varying *c* within biologically relevant ranges does not qualitatively affect our result (S7 Fig). Further note that the relative magnitudes of the decrease and subsequent increase of search times are smaller in this case compared to the uniform probability case. The power-law contact probability takes into account realistic polymer properties and is hence more reflective of search times that can be achieved in biological contexts.

This central result of the importance of polymer conformations is shown schematically in Fig 2c, along with illustrative simulation trajectories. For relatively open conformations, the kinetics is dominated by 1D sliding (blue segments), with very few intersegmental hops, resulting in large target search times. As the polymer becomes increasingly compact, distant segments come into contact, and these intersegmental hops lead to faster search. Conversely, for highly collapsed conformations, there is a preponderance of long hops (red segments), again increasing search times. In between these two extremes lies a regime of optimal compaction, where a judicious mix of 1D sliding and intersegmental hops leads to an optimisation of search times.

### 3.2 Role of bulk diffusion in target search within compact domains

The classical facilitated diffusion model posits that target search happens through repeated rounds of 1D sliding and bulk 3D diffusion, with an optimal unbinding rate that minimises search times. However, this formalism ignores the role of intersegmental jumps due to the polymer topology itself. We now ask whether bulk diffusion is an advantageous search strategy when we take into account the effects of the polymer compaction itself (see S2 Text). For a protein searching for targets through a combination of 1D sliding and intersegmental hops, the polymer compaction allows a route to minimize search times. How is this search strategy affected if we now consider the effect of bulk 3D diffusion, in addition to 1D sliding and intersegmental jumps? Can search times be further decreased by introducing bulk excursions? Our contact space representation of a polymer allows us to take into account the role of 3D diffusion ($p_{off}\neq 0$), with the assumption that rebinding times are long enough that the memory of the unbinding site is lost, and rebinding occurs randomly at any site along the polymer [49,50]. Experimental estimates of the 1D diffusion coefficients of proteins are in the range of 10^4^ − $10^{6}bp^{2}/s$. Unbinding rates for various proteins have also been reported in the range 10–2000*s*^−1^. Taken together, these imply unbinding probabilities for the searching protein in the range 10^−1^–10^−4^ for kinetics of TFs [11,15,68].

**Fig 3:**
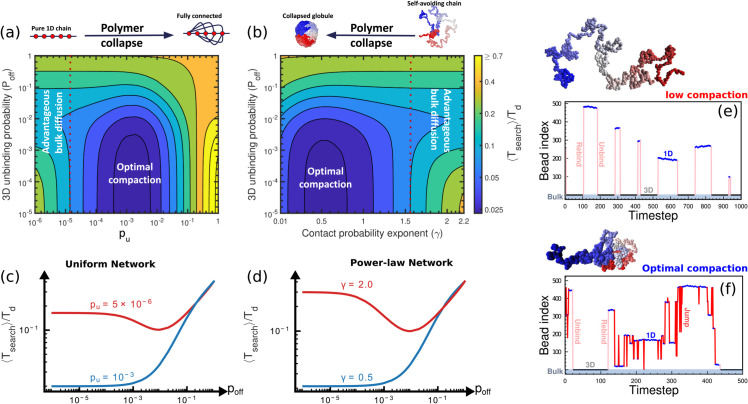
Optimal polymer compaction minimises search times even in presence of 3D diffusion. Contour plot of $\langle T_{search}\rangle /T_{d}$ as a function of 3D unbinding probability ($p_{off}$) and polymer compaction for **(a)** a uniformly connected domain and **(b)** a power-law connected domain of *L* = 500. In a relatively open chain (see left side in **(a)** and right side in **(b)**, there is an optimal $p_{off}$ for which $\left\langle T_{search}\right\rangle$ is minimum suggesting 3D excursion is advantageous for these conformations (red curves in **(c)** and **(d)**). However, as the polymer collapses, $\left\langle T_{search}\right\rangle$ monotonically decreases with decreasing $p_{off}$ suggesting that 3D excursions are not advantageous for compact polymers (blue curves in **(c)** and **(d)**). For biologically relevant $p_{off}$ regimes, there always exists an optimal polymer compaction where $\left\langle T_{search}\right\rangle$ is minimum. The fact that the global minimum of $\left\langle T_{search}\right\rangle$ occurs for highly folded conformations and in regions where unbinding is low suggests the role of 3D spatial structure of chromatin in the search process. In **(e)**, trajectory of protein for open polymer configurations (low *p*_*u*_ or, high *γ*) shows frequent detachment to bulk from the polymer and reattachment to the polymer. In **(f)**, trajectory of protein for polymer at optimal compaction shows rare unbinding from the polymer and frequent intersegmental jumps.

We first consider the case of γ=0 (Fig 3a), so that $P_{c}(s)=p_{u}$. As $p_{u}\to 0$, we recover the classical facilitated diffusion result of an optimum search at an intermediate value of $p_{off}$. This is also shown in the cross-section plot for $p_{u}=10^{-5}$ in Fig 3c where $p_{off}∼10^{-2}$ results in minimum search times. As *p*_*u*_ increases, the advantage obtained through bulk diffusion reduces, until beyond a certain threshold, unbinding from the backbone offers no advantage to the target search process. The regime of validity of this standard 1D+3D facilitated diffusion is surprisingly small, with only a few intersegmental bonds ($p_{u}>10^{-5}$ for *L* = 500) enough to disrupt this classical result. Beyond this threshold, within this random rebinding model, 3D unbinding is not advantageous for target search, with the search times increasing monotonically with increasing $p_{off}$ in this regime. This can also be seen from the cross-section plot for $p_{u}=10^{-3}$, showing bulk diffusion is not advantageous for compact domains (Fig 3c). Interestingly, our result of an optimal compaction minimising search times continues to hold true even in the presence of 3D diffusion, with $\left\langle T_{search}\right\rangle$ being minimised at an optimal connectivity (*p*_*u*_). This result holds true for all unbinding probabilities below $p_{off}≲0.1$, consistent with estimated physiological unbinding probabilities.

This general result holds true when we look at power-law connectivities (Fig 3b) as well. For the self-avoiding polymer (γ=2.2), 3D diffusion offers an advantage to target search. As the polymer collapses, and hence *γ* decreases, the relative speed-up through bulk excursions decreases. Beyond the RW polymer (γ∼1.5), unbinding from the backbone offers no advantage to the target search process, as intersegmental hops allow a more efficient mechanism to explore distant sites. Indeed for collapsed polymers (low *γ* or large *p*_*u*_), and in the vicinity of the optimal compaction state, remarkably, 3D diffusion is not helpful for target search, with a monotonic increase in search times with increasing unbinding probability (S8 Fig). This contrasting effect of bulk diffusion depending on the polymer configuration can also be seen in the cross-section plots for an open (γ=2.0) vs compact (γ=0.5) polymer in Fig 3d, showing a monotonic increase with increasing 3D unbinding for the compact polymer (S8 Fig). This central result is shown schematically in Fig 3e and 3f. Our qualitative results continue to hold true for a wide range of bulk residence times, $\tau_{f}$ (S9 Fig). For low compaction, bulk excursions are frequent, interspersed with 1D sliding, while for optimal compaction, there are very few bulk excursions, with a mixture of sliding and intersegmental hops dominating the kinetics of the search process.

### 3.3 Polymer models of chromatin identify the existence of optimal compaction

We now examine whether non-monotonic search times persist when canonical polymer models are used to model chromatin structure (see Sect 2). In contrast to the contact space representation of polymers, in this case the allowed intersegmental jumps arise directly from polymer topology, and hence random walks on these backbones have direct relevance to protein search processes. As polymers collapse, the contact probability $P_{c}(s)=cs^{-\gamma }$ changes, and this is reflected in a coupled change of both the pre-factor *c* and the exponent *γ*. Thus search processes on polymer backbones offer a pathway to study the effect of compaction on search times distinct from the contact space representation of polymers. A variety of equilibrium as well as non-equilibrium polymer models have been studied in the literature to understand chromatin organisation at different scales [34,69]. We restrict ourselves to three canonical polymer models - the freely rotating chain, the bead spring Lennard Jones polymer, and a soft Lennard Jones polymer. Having investigated the role of bulk diffusion for compact domains, we limit ourselves to target search by 1D diffusion and intersegmental hops on these polymer backbones.

**Fig 4:**
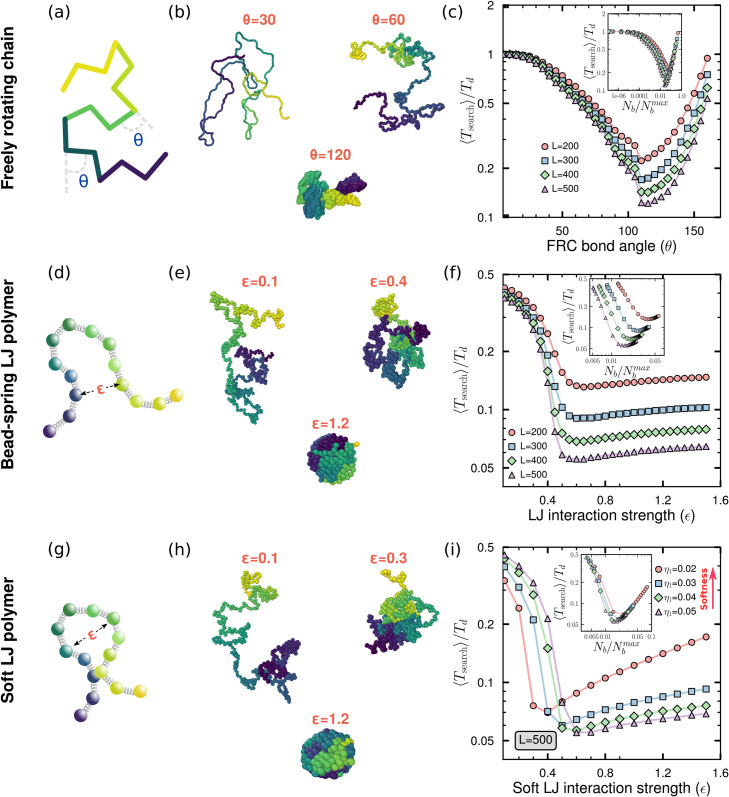
Search on polymer backbones reveals the existence of an optimal configuration that minimises search time. **(a)** Schematic of a FRC polymer (see Sect 2.5.1) with a bond angle *θ*. **(b)** Representative configurations of FRC polymers of length *L* = 500 at $\theta =30^{\circ },60^{\circ },120^{\circ }$, showing increasing compaction with increasing *θ*. **(c)** $\left(\langle T_{search}\rangle /T_{d}\right)$ in FRC polymers as a function of bond angle *θ*, and the scaled number of non-bonded contacts $N_{b}/N_{b}^{max}$ (inset) for *L* = 200–500. $N_{b}^{max}={\;}^{L}C_{2}$ − *L* denotes the maximum possible number of non-bonded contacts. **(d)** Schematic of a bead-spring polymer with attractive LJ interactions between non-bonded pairs (see Sect 2.5.2). **(e)** Representative configurations for polymers of length *L* = 500 at LJ interaction strengths (*ϵ*) = 0.1,0.4,1.2. Lower *ϵ* results in open conformations with higher *R*_*g*_ and lower non-bonded contacts, *N*_*b*_. Stronger *ϵ* lead to compact, globule-like structures with lower *R*_*g*_ and higher *N*_*b*_. **(f)** $\left(\langle T_{search}\rangle /T_{d}\right)$ in LJ polymers as a function of interaction strength *ϵ* and the scaled number of non-bonded contacts $N_{b}/N_{b}^{max}$ (inset) for *L* = 200–500. **(g)** In polymers with soft LJ interactions, bead overlap is permitted and controlled by a softness parameter $\eta_{1}$ (see Sect 2.5.3). Soft LJ polymers exhibit more compact configurations at higher *ϵ* compared to regular LJ polymers. **(h)** Representative configurations of soft LJ polymers of length *L* = 500 at ϵ=0.1,0.3,1.2. **(i)** $\left(\langle T_{search}\rangle /T_{d}\right)$ in soft LJ polymers as a function of *ϵ* and $N_{b}/N_{b}^{max}$ (inset) for *L* = 500, shown for different softness levels characterised by $\eta_{1}$.

We first study the freely rotating chain (FRC) model (see Sect 2.5.1) which has been used to investigate chromatin behaviour [53,54]. The FRC angle *θ* (Fig 4a) controls the compaction of the polymer, with large *θ* leading to a collapsed state (Fig 4b and S10 Figa). This simple model does not take into account excluded volume or other interactions. Nevertheless this can act as controlled model system from where to begin our investigation into the role of compaction from polymer topologies. The FRC bond angle introduces a persistence length *l*_*p*_ which decreases with increasing *θ*. The power-law scaling of the contact probability holds only for lengths above the persistent length, $l\gg l_{p}$. Further, the contact probability exponent does not depend on the FRC angle, instead the change in contact probability may be interpreted in terms of an increase in the prefactor *c* with increasing *θ* (see S10 Figc). As the polymer collapses, the number of allowed intersegmental jumps increases. We quantify the number of such allowed intersegmental contacts *N*_*b*_, which increases monotonically with *θ* (see S10 Figb). We simulated search on these FRC backbones, and the mean search time $\left\langle T_{search}\right\rangle$ shows a non-monotonic behaviour with increasing bond angle, and hence polymer compactness (Fig 4c), and also the number of intersegmental bonds (Fig 4c inset). Thus in line with the expectations from the network model of polymer, there exists an optimal compaction where search times are minimum.

We next use a bead-spring polymer model (Fig 4d) of chromatin with attractive Lennard-Jones (LJ) potential (see Sect 2.5.2) [70–72]. This introduces self-avoidance effects absent in the FRC model. With increase of the LJ interaction strength *ϵ*, the polymer undergoes a second-order phase transition from a coiled to a compact state (Fig 4e, and S11 Figa), accompanied by an increase in the number of possible intersegmental jumps (see S11 Figb). As the polymer collapses, the scaling exponent *γ* reduces from the self-avoiding exponent. Beyond the collapse transition, *γ* does not decrease further, even with increasing *ϵ*. For these compact conformations, the power-law description itself only holds at intermediate scales, with a saturation for very long range contacts [73,74] (see S11 Figc). The search times $\left\langle T_{search}\right\rangle$ on these LJ polymer backbones exhibited a non-monotonic pattern as the LJ interaction strength is increased (Fig 4f), or equivalently as the connectivity of the polymer increases (Fig 4f inset). There was a distinct minimum observed in the search times, occurring close to physiological chromatin compactions. Thus a LJ polymer qualitatively mirrors the results obtained from the uniform network and FRC polymer models.

However, unlike the significant rise seen in the FRC and the network model, the extent of $\left\langle T_{search}\right\rangle$ increase beyond a critical *ϵ* is less pronounced in this case. This limited increase is due to the inherent hard-core repulsion in the LJ potential, which imposes a lower limit on polymer compaction, and on the number of intersegmental bonds. Since *γ* saturates beyond the critical *ϵ*, this increase can also be interpreted in terms of a marginal increase in the total number of contacts at the same *γ*. This interplay between LJ interactions and polymer structure thus introduces a unique feature that distinguishes it from both FRC and the network model.

Is this weak non-monotonic rise in $\left\langle T_{search}\right\rangle$ then the correct physical description of search times? The answer hinges on whether the hardcore LJ potential best represents coarse-grained chromatin conformations. Experimental and theoretical studies of chromatin configurations and 3D distances suggest that a soft inter-bead potential that allows for overlap is a more realistic description of coarse-grained chromatin [28,75]. We study a polymer model (Fig 4g) with a soft inter-bead potential (see Sect 2.5.3, Fig 4h), with the softness controlled by the parameter $\eta_{1}$ (S12 Fig). We investigate $\left\langle T_{search}\right\rangle$ on such soft polymers with varying degrees of softness. A lower value of $\eta_{1}$ indicates a softer polymer, whereas high $\eta_{1}$ resembles the classical LJ potential. For a very soft polymer ($\eta_{1}=0.02$), $\left\langle T_{search}\right\rangle$ shows a strong non-monotonicity, analogous to the FRC and the network results (Fig 4i). This is because a soft polymer core cannot effectively enforce volume exclusion, resulting in a more tightly packed polymer compared to the conventional LJ model (see S13 Figa). This then leads to a higher number of non-neighbouring contacts within the polymer domain (see S13 Figb) and hence a stronger non-monotonicity in search times (Fig 4i inset). In terms of the contact probability, the initial decrease is then due to the change of the scaling exponent *γ*. Beyond the collapse transition, the soft potential allows for more monomers to come into contact, increasing the number of possible intersegmental hops, leading to the non-monotonic behaviour (see also S13 Figc). As we decrease the softness (increasing $\eta_{1}$), the degree of non-monotonicity in the search times decreases and approaches the LJ-like behaviour. Thus non-monotonic behaviour of search times remains a crucial feature of protein diffusion on coarse-grained chromatin-like soft polymer topologies. The degree of non-monotonicity in $\left\langle T_{search}\right\rangle$ is then controlled by the softness of the inter-bead potential.

### 3.4 Topological domains are self-organised near optimal compaction

**Fig 5:**
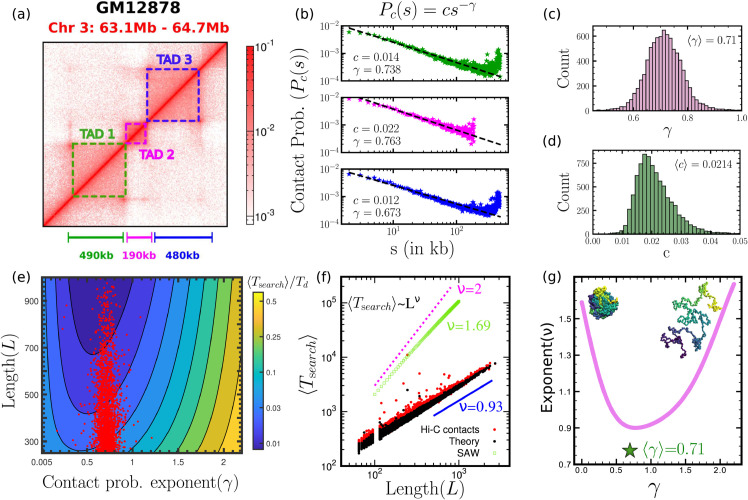
Experimentally identified TADs occupy the zone of optimal compaction and leads to ballistic search. **(a)** An example of 3 distinct TAD regions in Hi-C contact map from [42]. **(b)** Contact probability (*P*_*c*_(*s*)) as a function of genomic distance (*s*), fitted using $P_{c}(s)=cs^{-\gamma }$ for the three TADs in (a). **(c-d)** Distribution of best fit parameters *c* and *γ* for all 8355 collected TADs from GM12878 data [42]. **(e)** Contour plot of scaled mean search times as a function of *γ* and *L*. The fact that experimentally observed [42] *L* and *γ* values (red dots) fall very close to the minimum is yet another evidence that 3D chromatin structure is relevant in the search processes. **(f)** Search times as a function of TAD lengths show a very different scaling compared to purely 1D diffusive search (pink dashed line, ν=2.0). Search time computed from simulating walks on network with Hi-C contact probability (red dots) and network with observed power-law properties (black) show $\langle T_{search}\rangle ∼L^{0.93}$. The observed exponent is very different from the target search time scaling on self avoiding walk (SAW) polymer indicating role of distinct chromatin organisation in search processes. **(g)** Scaling exponent (ν) of $\left\langle T_{search}\right\rangle$ with domain length *L* for different contact probability scaling exponent *γ*. ν is higher for both open (γ=2.2) and compact polymer (γ<0.5) configuration and shows a minimum near the experimentally observed *γ* range of TAD, marked by green star.

Is this non-monotonic behaviour of search times relevant for real TADs? Where do TAD conformations lie with respect to this optimal compaction state? How fast is the search process for optimal chromatin compaction? To answer these, we turn to publicly available experimental Hi-C contact probability data [42] and perform the following theoretical study (see Sect 2). We identify 8355 TADs across all chromosomes from the GM12878 cell line (see Fig 5a and S14 Figb) and analyse Hi-C data of these TADs at 1 kbp resolution. To characterise each TAD’s spatial organisation, we quantify the contact probability as a function of genomic separation, *P*_*c*_(*s*) obtained from the normalised Hi-C frequency matrix. The identified TADs have a broad distribution of lengths ranging from 65 kbp - 2600 kbp (see S14 Figc). For each TAD of genomic length *L*, we fit the contact probability curve to a power-law $P_{c}(s)=cs^{-\gamma }$ over a range *s* = 10 to (*L*–100) kbp, as shown in Fig 5b. This allows us to quantify two parameters - the contact probability scaling exponent *γ*, and the prefactor *c*, for each individual TAD. We then analyse the distributions of the fitted values of *c* and *γ* across all TADs, as shown in Fig 5c and 5d, which yield average values of ⟨c⟩=0.0214 and ⟨γ⟩=0.71. Our estimate of ⟨c⟩ is also consistent with values which reproduce experimentally known mean spatial distances between genomic loci [28]. Both the contact probability exponent and the prefactor are independent of the size of the TAD (see S14 Figd). The most probable values of *γ* fall within the range of approximately 0.66 to 0.75, indicating a consistent degree of compaction among these TADs, and implying that topological domains are more compacted than the overall chromosome organisation, which has an average contact probability exponent γ≃1.08 [39]. We first compute protein target search times across a broad spectrum of domain lengths *L* and contact probability exponent values *γ* on a power-law network with *c* fixed at its average value, ⟨c⟩. The resulting search time estimates are represented as a contour map in Fig 5e in the (*L*,*γ*) phase plane. To assess how these findings relate to optimal search efficiency in TADs, we overlay the fitted *γ* values and corresponding lengths *L* for each individual TAD onto the contour map in Fig 5e, as indicated by red dots. Strikingly, we observe that the TAD conformations cluster near regions of the contour map corresponding to minimal search times, irrespective of the length of the TAD, facilitating rapid target finding and potentially enhancing genomic function. This clustering of TADs near the optimal region is insensitive to variations of the prefactor *c* over the biologically relevant range (Fig 5d), as is shown in S7 Fig.

To further investigate the impact of chromatin organisation at the TAD scale on target search, we now turn to the question of how fast is the target search process on these optimally compacted chromatin conformations? To address this, we generate chromatin networks by employing two distinct methodologies (see Sect 2.6 for details). First, we construct *power-law networks* for each Topologically Associating Domain (TAD) of length *L* using parameters *c* and *γ* from fitting the power-law distribution $P_{c}(s)=cs^{-\gamma }$ to Hi-C data. This method allows us to simulate chromatin networks with averaged contact probabilities, and hence spatial relationships, which mimic the conformations of real topological domains in chromatin without requiring detailed region-specific contacts. In the second approach, we created direct Hi-C networks based on experimentally measured contact probabilities *P*_*ij*_ between chromatin loci *i* and *j* within each TAD (S14 Figa). Unlike the power-law method, this approach incorporates region specific contact probabilities obtained from Hi-C data, thus preserving detailed sequence-specific interactions within chromatin. This method allows us to capture the unique spatial organisation within each TAD and explore how these specific contacts impact the search dynamics. The mean search times on these networks are shown in Fig 5f. Interestingly, we find that the search times scale with TAD lengths in a near-ballistic manner, following $\langle T_{search}\rangle ∼L^{0.93}$. This near-ballistic scaling suggests that the organisation within these TADs enables target search to proceed in an effectively driven fashion, even though the underlying dynamics is purely diffusive. For pure one-dimensional sliding along the polymer contour, one would expect $\langle T_{search}\rangle ∼L^{2}$. However, intersegmental hops change this scaling of search times to yield a contact probability dependent scaling, $\langle T_{search}\rangle ∼L^{\nu (\gamma )}$. This effective exponent ν(γ) also shows a minimum around the TAD range (Fig 5g), with larger ν values both for collapsed globule, and random walk or self-avoiding polymers. Our findings imply that random walks on these power-law-structured TADs can significantly accelerate search processes, offering a large speed-up relative to purely diffusive timescales (where $\langle T_{search}\rangle ∼L^{2}$). This efficiency appears to result from the inherent structure of TADs rather than from any additional motor or energy-dependent mechanisms, as illustrated in Fig 5f. Moreover, we find that this result is consistent in both the approaches - power-law networks, and Hi-C based contacts, suggesting that although Hi-C probability-based networks capture detailed region-specific effects, in the context of search processes, power-law models offer an accurate approximation of search times expected for biological search processes on chromatin. Thus, these models provide a robust framework for estimating protein dynamics and highlight the importance of TAD organisation in optimising genomic search efficiency.

## 4 Discussion and conclusion

We investigate a stochastic model of protein dynamics that integrates experimental Hi-C data and hence the role of the 3D structure of chromatin in connection to target search. We propose that facilitated diffusion which takes into account intersegmental jumps can leverage the compaction state of chromatin, characterised by the presence of TADs and looped polymer conformations, to access distant genomic regions for more efficient search. This is in line with direct evidence from single-molecule experiments which have reported that search process by a site-specific restriction enzyme on DNA becomes almost doubly efficient when the DNA configuration is collapsed instead of a fully extended configuration [76]. Remarkably, contrary to the prevailing picture of bulk diffusion mediated search, we show that for highly folded polymer conformations, such as in chromatin, bulk diffusion hinders the search process, possibly offering a reconciliation with experimental estimates of very large times a protein spends non-specifically bound to DNA. An analysis of contact probabilities from experimentally determined Hi-C data suggests that TADs reside near this zone of optimal compaction where search times are minimised. Target search on these TAD structures can thus facilitate rapid dynamics of DNA-associated proteins, which is crucial for gene regulation and cellular function.

### 4.1 Strengths and limitations of the model

Our theoretical analysis, for the first time, investigates the role of chromatin conformations itself as a control parameter in regulating search times. The strength of our analysis lies in the fact that we use experimental contact probability data from Hi-C experiments to understand how intersegmental contacts at the TAD scales shapes search times. Although there has been significant attention on the connection between gene expression and enhancer-promoter interactions facilitated by chromatin looping, the role of the broader 3D chromatin architecture on protein search remains largely unexplored. By dissecting the sub-TAD organisation, we aim to bridge the gap in understanding how chromatin’s 3D structural features contribute to the efficiency of protein search processes within the genome. While our analysis focuses on relatively short genomic regions (up to 2 Mbp), the methodology we employ is quite general and can be extended to larger scales. However, our contact space network representation of a polymer makes simplifying assumptions about the polymer conformations. In particular it neglects higher order correlation due to the looped structures [77]. Such correlations are not expected to play a major role for dynamic polymers [44,78], assuming that the search timescales are larger than the timescales of polymer dynamics, and this allows us to use this representation to calculate search times. Further, using Hi-C data also has its inherent limitations. Hi-C contact matrices represent time-averaged contact frequencies generated from millions of reads, and how this translates to real chromatin conformation is still an open question. Hi-C contact matrices also measure relative probabilities, and does not determine the prefactor in the scaling relation. However, our choice of the prefactor *c* has been shown to yield sensible estimates of 3D distances between genomic loci [28], and hence are expected to correspond to biologically relevant ranges. Finally, while our analysis focuses solely on the role of the chromatin conformation, other biological factors may also influence target search times. These include the role of epigenetic modifications, as well as dynamic processes such as loop extrusion, nucleosomal kinetics and breathing which affect local polymer properties [79]. However, the timescales of protein search, which is much faster than loop extrusion [80], and the 1 kbp resolution, which is larger than nucleosomal lengthscales [28], allows us to ignore these dynamic processes in our analysis. Note that our 1 kbp coarse-graining also implies that for bulk diffusion, we neglect very fast local rebinding, as these are absorbed into the effective residence time on the coarse-grained beads. We also neglect any cooperative effects or interactions between proteins, or recruitment of transcription factors due to interactions with other protein machineries, such as within transcription factories. Nevertheless, our analysis highlights how regulating polymer conformations alone can result in faster search by a factor of 10–50 compared to target search on extended conformations.

### 4.2 Suggestions for new experiments to test our predictions

Our key prediction is that chromatin structure affects target search. To test this prediction, one can generate DNA or chromatin configurations with different structures having different compactions. One possible way to achieve is this is to insert multiple artificial CTCF binding sequences in the genome so that more looped structures are formed. One can engineer (either within the genome or in a long plasmid outside the chromosomes) the location and direction of these boundary sequences such that a 3D structure of a given contact probability exponent *γ* may be achieved. One can measure the statistics of protein reaching a well-defined sequence-specific target on this structure. Another way to assemble chromatin structure of varying compaction is to assemble DNA/chromatin with varying concentrations of DNA-compacting proteins like HP1 or other architectural proteins [81–83], as well as salt concentration to tune detachment events in vitro [76]. Systematic experiments can then be designed to explore how these changes affect protein dynamics and hence search times. Recent advances in single molecule tracking experiments also allow us to study the dynamics of TFs over long time scales [84]. Visualisation of a pluripotency factor Sox2 inside a topological domain observed distinct hopping behaviour which suggested that chromatin domains sequester proteins and kinetically facilitates search, consistent with the results of our analysis. Systematic tracking of TF motion in different topological domains with varying levels of compaction using such single particle techniques can further help validate our hypothesis.

### 4.3 Outlook and relevance to chromatin organisation and function

The hierarchical organisation of chromatin in mammalian cells, from chromosome territories at scales of ∼10 Mbp to topologically associating domains at hundreds of kilobases has now been recognised from various experiments [42]. This scale dependent organisation implies that search strategies by proteins may also need to be suitably modified at these different scales. While bulk diffusion in the nucleoplasm can be effective in locating the right spatial domain for a particular protein, target search at the scale of TADs requires leveraging chromatin contacts into intersegmental hops. Crucially, intersegmental transfers, which depend on the topology of the polymer, and hence introduces correlated jumps offers a distinct mechanism from bulk diffusion. Indeed, our analysis shows that search processes which leverage allowed intersegmental hops are generally much faster than those that proceed through a combination of 1D sliding and 3D diffusion. Target search inside the nucleus through intersegmental transfers has consequences not only for gene expression via transcription factor binding, but also plays a role in DNA damage repair, where repair proteins need to locates the site of DNA breaks [85]. Our findings highlight the critical role chromatin structure plays in influencing the search dynamics of nuclear proteins, and is consistent with emerging experimental data from a range of diverse experiments [76,84,86].

## Supporting information

S1 Text Protein motion in coarse-grained chromatin. (PDF)

S2 Text Theoretical estimation of mean search times. (PDF)

S1 Fig In a dense chromatin environment, proteins remain in prolonged contact with the coarse-grained polymer. **(a)** Schematic of the construction of a coarse-grained chromatin polymer, where each bead represents 1 kb, obtained from fine-grained nucleosome beads (200 bp). Short-range hopping events appear as intersegmental jumps in the coarse-grained representation. **(b)** Residence probability in 3D diffusion mode, where the protein is not bound to chromatin, varies as a function of the volume fraction of chromatin in the box. As the volume fraction increases, bulk exploration decreases due to the limited space for free movement in densely packed chromatin regions. **(c)** Representative protein trajectories illustrating the motion at volume fractions of 1% and 10%. At low volume fractions, motion is primarily governed by binding and unbinding processes. However, at high volume fractions, the motion exhibits numerous intersegmental jumps.
(TIFF)

S2 FigNon-monotonic behaviour of search times remains robust under network rewiring and different slide/jump timescales.**(a)** $\frac{\left\langle T_{search}\right\rangle }{T_{d}}$ in uniformly connected network domain vs *p*_*u*_ for different dynamic rewiring for *L* = 100. The non-monotonic behaviour of $\left\langle T_{search}\right\rangle$ remains robust even when the polymer network undergoes dynamic re-configurations at a fixed connection probability. Dynamic rewiring of network connections reduces $\left\langle T_{search}\right\rangle$, with faster configuration changes yielding lower $\left\langle T_{search}\right\rangle$ at low *p*_*u*_ values. However, at high *p*_*u*_ values, network dynamicity offers little advantage, as most nodes already have numerous long-range connections. **(b)** $\frac{\left\langle T_{search}\right\rangle }{T_{d}}$ in uniformly connected network domain vs *p*_*u*_ for different jump times for *L* = 100. We show that the non-monotonic behaviour of $\left\langle T_{search}\right\rangle$ persists even when sliding along the backbone and jumps along intersegmental bonds have different timescales. At low *p*_*u*_, the $\left\langle T_{search}\right\rangle$ remains independent of $t_{jump}$, dominated by 1D sliding. Conversely, for higher *p*_*u*_, where jumps are more probable than sliding, $\left\langle T_{search}\right\rangle$ increases as the $t_{jump}$ increases.(TIFF)

S3 FigThe optimal behaviour of $\left\langle T_{search}\right\rangle$ is consistent for all polymer lengths (*L*).Scaled mean search time in a uniformly connected domain as a function of *p*_*u*_ and domain lengths (*L*). The colour bar represents scaled mean search time $\frac{\left\langle T_{search}\right\rangle }{T_{d}}$.(TIFF)

S4 FigSearch times are non-monotonic irrespective of protein start positions.Scaled mean search time $\left(\frac{\left\langle T_{search}\right\rangle }{T_{d}}\right)$ for different initial positions for **(a)** uniformly connected network and **(b)** power-law connected network domain for different initial position for L=500.(TIFF)

S5 FigAn effective medium approximation reproduces search times near fully connected polymer networks.Scaled mean search time $\left(\frac{\left\langle T_{search}\right\rangle }{T_{d}}\right)$ in a uniformly connected network domain vs probability of contact (*p*_*u*_) between any two non-neighbouring beads for *L* = 100. Simulation results are shown as circles, and the effective medium prediction is shown as a blue line.(TIFF)

S6 FigNon-monotonic search times are robust for different levels of boundary insulation.The $p_{u}^{\prime }=0$ case corresponds to complete insulation, where the boundary sites do not have any links to the interior sites, corresponding to the current figures in the manuscript. We now study two additional cases, where the boundary site can have a link to any interior site with a 100-fold reduction in probability ($p_{u}^{\prime }=0.01p_{u}$), and where the boundary site can have a link to any interior site with a 10-fold reduction in probability ($p_{u}^{\prime }=0.1p_{u}$). In the first case ($p_{u}^{\prime }=0.01p_{u}$), we almost recover $p_{u}^{\prime }=0$ result, showing that a 100-fold insulation is enough for this optimisation of search times. Even in the 10-fold reduction, which is very weak insulation, the qualitative nature of our results continues to hold.(TIFF)

S7 FigThe position of the minimum in search times curve does not change drastically across biologically relevant ranges of *c* values.The scaled mean search time, $\left(\frac{\left\langle T_{search}\right\rangle }{T_{d}}\right)$, in a power-law connected network with contact probability $P_{c}(s)=cs^{-\gamma }$, is plotted as a function of the exponent *γ* for a polymer of length *L* = 500. The mean search time exhibits a consistent non-monotonic dependence on *γ* as the prefactor *c* is varied within the biologically relevant range inferred from Hi-C data.(TIFF)

S8 FigAverage search time as a function of polymer compaction for different $p_{off}$.Average search time as a function of polymer compaction is shown for varying unbinding probabilities $p_{off}$ in **(a)** uniformly connected domains and **(c)** power-law connected domains. Conversely, average search time as a function of $p_{off}$ for varying levels of polymer compaction is shown in **(b)** uniformly connected domains and **(d)** power-law connected domains. When the unbinding probability is low, increased polymer compaction can facilitate intersegmental transfer, thereby reducing search times. In this regime, an optimal compaction exists at which the search time is minimised. However, at high $p_{off}$, the polymer architecture becomes irrelevant, resulting in approximately constant search times across different compaction levels (see panels (a) and (c)). On the other hand, when the polymer is less compact (i.e., low *p*_*u*_ or high *γ*), an optimal unbinding probability emerges that minimises the search time. This non-monotonic dependence on $p_{off}$ disappears at higher compaction levels, a regime more representative of TAD-like chromatin domains (see panels (b) and (d)).(TIFF)

S9 FigAverage search time as a function of polymer compaction for different rebinding times.Average search time as a function of polymer compaction for different unbinding probabilities ($p_{off}$) in uniformly connected domains and power-law connected domains, shown for three rebinding times: $\tau_{f}=10\tau ,100\tau ,$ and 1000τ. For both uniform and power-law networks, for an open polymer ($p_{u}\to 0,\gamma \to 2.2$), search via 3D diffusion is faster than search through intersegmental transfers. For these open polymers, there is an optimum $p_{off}$ which minimises search times, consistent with canonical facilitated diffusion. However, as the connectivity increases, search via intersegmental transfers becomes faster, and search times increase with increasing $p_{off}$. For uniform networks, beyond a certain connectivity, 3D diffusion becomes faster again for these small *p*_*u*_ values, however, for power-law networks, search via intersegmental transfers remains the optimal strategy even till very low values of *γ*. At optimal polymer connectivity, search via intersegmental transfer is more advantageous than 3D diffusion, even for fast rebinding.(TIFF)

S10 Fig Radius of gyration, number of intersegmental contacts and contact probability for FRC polymer. **(a)** Radius of gyration (*R*_*g*_) as a function of FRC bond angle (*θ*). **(b)** Number of intersegmental contacts (*N*_*b*_) as a function of *θ*. The angle *θ* controls the compaction of the polymer, with large *θ* leading to a collapsed state. As the polymer collapses, the number of non-neighbouring bonds *N*_*b*_ increases monotonically, indicating a greater probability of intersegmental jumps. **(c)** Contact probability as a function of *s* for different *θ*.
(TIFF)

S11 Fig Radius of gyration, number of intersegmental contacts and contact probability for LJ polymer. **(a)** Radius of gyration (*R*_*g*_) as a function of LJ interaction strength (*ϵ*). **(b)** Number of intersegmental contacts (*N*_*b*_) as a function of LJ interaction strength (*ϵ*). A LJ polymer undergoes a transition from an open to a compact state, accompanied by an increase in the number of intersegmental bonds, as *ϵ* is increased. **(c)** Contact probability as a function of *s* for different LJ interaction strength (*ϵ*).
(TIFF)

S12 FigNature of the soft LJ potential for different softness.$\eta_{1}$ controls the softness of the polymer. As $\eta_{1}$ is decreased, the softness of the polymer increases. In the inset, zoomed view of the potential near minima. The expression *V*_0_ − *ϵ* denotes the energy penalty associated with complete overlap, and the parameters $\eta_{1}$ and $\eta_{2}$ are utilised to adjust the softness of the potential. When *r* < *r*_*m*_, the potential becomes repulsive but with a softer behaviour compared to the LJ potential. For $r_{m}\leq r<2.5\sigma$, the potential aligns with the LJ potential, inducing attractive interactions. Beyond a cutoff distance (r≥2.5σ), the interaction energy is set to zero. Parameters of the potential for our study: $V_{0}=10^{7}$, $\eta_{1}=\text{variable}$, $\eta_{2}=3.16$, *r*_*m*_ = 1.12, σ=1.(TIFF)

S13 FigRadius of gyration, number of intersegmental contacts and contact probability for soft-LJ polymer.**(a)** Radius of gyration (*R*_*g*_) as a function of soft LJ interaction strength (*ϵ*) for $\eta_{1}=0.02,0.03,0.04,0.05$ for *L* = 500. **(b)** Number of intersegmental contacts (*N*_*b*_) as a function of soft LJ interaction strength (*ϵ*). **(c)** Contact probability as a function of *s* for different soft LJ interaction strength (*ϵ*) at $\eta_{1}=0.02$.(TIFF)

S14 Fig Genome-wide statistics of quantities used in this study across all analysed TADs. **(a)** A representative topologically associating domain (TAD) identified from human Hi-C data, where the contact probability between any two genomic segments *i* and *j* is denoted by *P*_*ij*_. An ensemble of networks is generated using this Hi-C data, where a contact between nodes *i* and *j* is established probabilistically according to the experimentally measured Hi-C contact probabilities *P*_*ij*_. To model the dynamics of a transcription factor (TF) within a realistic chromatin environment, we employ a previously established model incorporating sliding and intersegmental jumps on a network. This network represents the chromatin structure and captures 3D spatial contacts within a single TAD, as derived from Hi-C data. **(b)** Number of TADs analysed for each human chromosome. **(c)** Distribution of TAD lengths across all analysed chromosomes. **(d)** Variation in the fitted parameters *c*, *γ*, and length *L* across all studied TADs.
(TIFF)
