# Supplementary material for: Compaction of chromatin domains regulates target search times of proteins
Source: PLoS Comput Biol. 2026 Jan 20;22(1):e1013843. doi: 10.1371/journal.pcbi.1013843 (PMC12858080; doi:10.1371/journal.pcbi.1013843)
Supplement: S1 Text — (PDF) [file pcbi.1013843.s001.pdf]

S1 Text. Protein motion in coarse-grained chromatin

Proteins within the nucleus search for their specific target sites and bind to them. These target sites usually consist of short sequences spanning only a few base pairs. In computational modelling, chromatin is often coarse-grained to a lower resolution, where each bead represents multiple base pairs of DNA. When modelling the search process in coarse-grained chromatin, the description of search strategies must be adjusted to reflect the level of coarse-graining. A key aspect of interest is the ratio of the time a protein spends bound to chromatin versus freely diffusing in the bulk nuclear environment.

While the widely studied facilitated diffusion model suggests that 1D motion along a chain accompanied by 3D bulk exploration is an efficient search strategy, bulk diffusion may not be a viable description for motion on coarse-grained chromatin at physiological chromatin volume fractions. The facilitated diffusion model is proposed for prokaryotic systems, where the density of chromatin is relatively low. In eukaryotic cells, the volume fraction of chromatin is, on average, 10%. However, chromatin is not uniformly distributed in the nucleus, and the local volume fraction within Topologically Associating Domains (TADs) can exceed the genome-wide average. Indeed, some experimental studies have shown extremely high chromatin volume fractions (50-60%) within $100 - 200$nm domains ([3, 1]). In an interphase cell, the mean volume fraction was estimated to be $30 \pm 10\%$ [1]. The high local density of chromatin restricts the bulk exploration of proteins, and hence the protein stays non-specifically bound to DNA for long periods of time. Further, the coarse-grained description of chromatin means that a unit bead contains a few kilobase pairs (kbp) of DNA, and the bead size becomes of the order of $10 - 100$ nanometers. Coarse-grained beads are also prone to overlapping. Therefore, the 3D diffusion at the base pair level is subsumed into the effective 1D motion in the coarse-grained picture.

In this section, we aim to determine the upper limit of the ratio between the time a protein spends freely diffusing in the bulk nuclear environment and the time it spends bound to chromatin. To this end, a bead-spring polymer model consisting of 3000 beads within a variable-sized box, allowing us to control the volume fraction of the system. Each bead in the polymer represents a ~ 200bp chromatin segment, which is the experimentally known smallest unit of chromatin. The protein is represented as a single particle moving randomly within the box, with interactions between all beads (protein and polymer) set to be purely self-avoiding (WCA). We conduct simulations of this system using LAMMPS[2] and collect trajectories of the protein. Upon obtaining the trajectories, we coarse-grain the polymer using the following strategy: we consider five consecutive beads along the polymer chain and calculate the end-to-end vector for this segment. We then place a coarse-grained bead at the center of mass (COM) of these five beads, with a diameter equal to the calculated end-to-end distance of this segment. Subsequently, we determine the distance of the protein from the center of each coarse-grained bead. If the protein is within a cutoff radius from center of a coarse-grained bead, we consider it to be bound to the chromatin. Otherwise, we consider the protein to be freely moving in the bulk nuclear environment.

In S1 Fig, we plot residence probability in 3D diffusion mode i.e. when the protein is not bound to chromatin as a function of the volume fraction of the chromatin in the box. As the volume fraction is increased, the bulk exploration is decreased due to lack of space for free movement in a highly dense chromatin region. This indicates, even if there is possibility that protein is following 1D+3D search strategy, that would not show up significantly while modelling the search process using a coarse-grained description. The motion of a protein can be purely modelled as a combination of slides and intersegmental jumps in such scenario. It is also important to note, in our analysis, we have not considered any specific or non-specific attractive interactions between the protein and the chromatin polymer. Additionally, we have not taken into account the structural constraints imposed by the chromatin polymer itself. In reality, both of these factors would further limit the protein's bulk exploration. For example, specific interactions between the protein and the chromatin polymer could

result in the protein preferentially binding to certain regions of the chromatin, reducing its overall exploration of the bulk. Similarly, the structural constraints of the chromatin polymer, such as loops and domains, would create barriers that the protein would need to navigate around, further limiting its exploration. Overall, the plot shows an estimate of upper limit of 3D diffusion probability in compact TAD-like domains.

References

[1] Horng D Ou et al. "ChromEMT: Visualizing 3D chromatin structure and compaction in interphase and mitotic cells". In: *Science* 357.6349 (2017), eaag0025.

[2] A. P. Thompson et al. "LAMMPS - a flexible simulation tool for particle-based materials modeling at the atomic, meso, and continuum scales". In: *Comp. Phys. Comm.* 271 (2022), p. 108171. DOI: 10.1016/j.cpc.2021.108171.

[3] Ranya KA Virk et al. "Disordered chromatin packing regulates phenotypic plasticity". In: *Science advances* 6.2 (2020), eaax6232.
