# Supplementary material for: Compaction of chromatin domains regulates target search times of proteins
Source: PLoS Comput Biol. 2026 Jan 20;22(1):e1013843. doi: 10.1371/journal.pcbi.1013843 (PMC12858080; doi:10.1371/journal.pcbi.1013843)
Supplement: S2 Text — (PDF) [file pcbi.1013843.s002.pdf]

S2 Text. Theoretical estimation of mean search times

S2.1 Matrix inversion to get mean search times

The network model can be represented using a set of equations that can be solved using matrix inversion. We can formulate the target search process as a mean first passage time problem for the protein to reach the target sites at $0, L$ for the first time. The protein walking on the domain can unbind from any bead with a probability p_{off} and rebind to any site including the boundary with a probability $\frac{1}{L+1}$ after spending a time τ_f diffusing in the bulk. At any site i, in addition to the nearest neighbours, protein can hop to 3D neighbours with a probability

$$P_c(s = |i-j|) = c|i-j|^{-\gamma} \quad \forall\, i, j \in [1, L-1]$$

When the walker is at position i the dynamic average of possible connections

$$N_i = 2 + \sum_{j \neq nn, 0, L} c|i-j|^{-\gamma} \quad \forall\, i, j \in [1, L-1]$$

Assuming that the time to perform a slide/jump to the neighbouring bead is τ, we can write the equation for average search time for a walker in the following matrix form

$$\mathbb{T} = \mathbb{A}^{-1}\mathbb{B}$$

where,

$$\mathbb{A}_{ij} = \begin{cases} \delta_{i,j} - \dfrac{1-p_{\text{off}}}{N_i}\big[\delta_{i\pm1,j} \\ \quad + (1-\delta_{i\pm1,j}-\delta_{f,j})(1-\delta_{0,j}-\delta_{L,j})c|i-j|^{-\gamma}\big] - p_{\text{off}}\delta_{f,j}, & \text{if } i \neq f, 0, L \\ \delta_{i,j} - (1-\delta_{i,j})\dfrac{1}{L+1}, & \text{if } i = f \\ \delta_{i,j}, & \text{if } i = 0, L \end{cases}$$

$$\mathbb{B}_i = \tau + (\tau_f - \tau)\delta_{i,f} - \tau(\delta_{i,0} + \delta_{i,L})$$

S2.2 Closed form solution for $p_{\text{off}} = 0$

In the case where protein's motion is purely along the chromatin backbone i.e. unbinding probability $p_{\text{off}} = 0$, we can rewrite a master equation for T_i–

$$T_i = \tau + r_s T_{i+1} + r_s T_{i-1} + \sum_{j \neq i, i+1, i-1} r_h T_j \tag{S1}$$

Here j is summed over all other possible non-neighbouring beads excluding boundaries$(0, L)$. τ is the time taken for a single step. As the walker takes no time to search if it starts from $0, L$, the absorbing boundary conditions for T_i is given by-

$$T_0 = 0 \quad \text{and} \quad T_L = 0$$

In the bulk, $i \in (1, L-1)$, the number of allowed intersegmental jumps (excluding the two nearest neighbours and both the boundaries) is $L-4$. Thus, normalisation conditions for slide and jump probabilities are given by–

$$2r_s + \sum_{\substack{i'=1 \\ i' \neq i, i\pm1}}^{L-1} r_h = 2r_s + (L-4)r_h = 1 \quad \forall i \in (1, L-1)$$

Using these relations, we can calculate explicit values of r_s and r_h as a function of p_u for a given L in the domain $i \in (1, L-1)$,

$$r_s = \frac{1}{2 + p_u(L-4)} \qquad\qquad r_h = \frac{p_u}{2 + p_u(L-4)}$$

At $i = 1, L-1$, the number of allowed intersegmental jumps (excluding the two nearest neighbours and the opposite boundary) is $L-3$. Thus, the slide and jumps probabilities are denoted as r'_s and r'_h respectively and normalisation conditions are given by,

$$2r'_s + \sum_{\substack{i'=2 \\ i' \neq i \pm 1}}^{L-1} r'_h = 2r'_s + (L-3)r'_h = 1 \qquad\qquad \text{if} \quad i = 1$$

$$2r'_s + \sum_{\substack{i'=1 \\ i' \neq i \pm 1}}^{L-2} r'_h = 2r'_s + (L-3)r'_h = 1 \qquad\qquad \text{if} \quad i = L-1$$

which gives,

$$r'_s = \frac{1}{2 + p_u(L-3)} \qquad\qquad r'_h = \frac{p_u}{2 + p_u(L-3)}$$

We construct a differential equation for the problem in the domain $\Delta x < x < (L-1)\Delta x$, where Δx is the spacing between two adjacent nodes and L is the total number of nodes starting from zero. The near boundary deviance will be introduced into the closed-form solution using appropriately defined boundary conditions.

To make the summation term uniform for all i values we add and subtract $r_h(T_{i-1} + T_i + T_{i+1})$ to the master equation Eq. (S1) to obtain-

$$(1 + r_h)T_i = \tau + (r_s - r_h)(T_{i+1} + T_{i-1}) + r_h \sum_{j=1}^{L-1} T_j$$

This can be rewritten as,

$$(1 + r_h)T_i - (r_s - r_h)(T_{i+1} + T_{i-1}) - r_h \sum_{j=1}^{L-1} T_j = \tau$$

The central difference formulation for the second derivative is given by-

$$\frac{\partial^2 T}{\partial x^2} = \frac{T_{i+1} - 2T_i + T_{i-1}}{(\Delta x)^2}$$

To introduce this form into our equation we add and subtract $2T_i$ to the second term hence giving us-

$$(1 + r_h)T_i - 2(r_s - r_h)T_i - (r_s - r_h)(T_{i+1} - 2T_i + T_{i-1}) - r_h \sum_{j=1}^{L-1} T_j = \tau$$

By multiplying and dividing the second term by Δx^2,

$$(1 + 3r_h - 2r_s)T_i - (r_s - r_h)\frac{(T_{i+1} - 2T_i + T_{i-1})}{\Delta x^2}(\Delta x^2) - (\tau + r_h \sum_{j=1}^{L-1} T_j) = 0$$

Assuming the given equation is in the finite difference form reverting it to the differential form while substituting $\Delta x = 1$ evaluates to-

$$(r_s - r_h)\frac{\partial^2 T[x]}{\partial x^2} - (1 + 3r_h - 2r_s)T[x] + (\tau + r_h \sum_{x=1}^{L-1} T[x]) = 0 \tag{S2}$$

We will concentrate on obtaining a closed-form solution for this equation within the bounds $0 < p_u < 1$ which says $(r_s - r_h) \neq 0$. We can rearrange the problem into the form-

$$\frac{\partial^2 T[x]}{\partial x^2} - \left(\frac{1 + 3r_h - 2r_s}{r_s - r_h}\right) T[x] + \left(\frac{\tau + r_h \sum_{x=1}^{L-1} T[x]}{r_s - r_h}\right) = 0 \tag{S3}$$

Since the summation term is independent of x we can prudently use the substitution:

$$\alpha = \frac{1 + 3r_h - 2r_s}{r_s - r_h} \qquad \beta = \frac{\tau + r_h \sum_{x=1}^{L-1} T[x]}{r_s - r_h}$$

to rewrite Eq. (S3) as-

$$\frac{\partial^2 T}{\partial x^2} - \alpha T + \beta = 0$$

The general solution of a differential equation of this form is given by-

$$T[x] = \frac{\beta}{\alpha} + C_1 e^{x\sqrt{\alpha}} + C_2 e^{-x\sqrt{\alpha}} \tag{S4}$$

Where C_1 and C_2 are constants of integration, we will use the boundary conditions to determine values of these constants.

Condition 1: $T[x]$ as a function of x depending on the value of p_u peaks at $x = \frac{L}{2}$ and going to zero at the boundaries. We can safely assume that the first derivative of the function $T[x]$ with respect to x goes to zero at $\frac{L}{2}$. Taking the first derivative of $T[x]$ from Eq. (S4) and substituting the condition gives-

$$\Rightarrow \frac{\partial T[L/2]}{\partial x} = \sqrt{\alpha}\left(C_1 e^{\frac{\sqrt{\alpha}L}{2}} - C_2 e^{-\frac{\sqrt{\alpha}L}{2}}\right) = 0$$

from the above equation we can derive $\left[C_2 = C_1 e^{\sqrt{\alpha}L}\right]$ and substitute in Eq. (S4) to get-

$$T[x] = \frac{\beta}{\alpha} + C_1(e^{x\sqrt{\alpha}} + e^{(L-x)\sqrt{\alpha}}) \tag{S5}$$

Condition 2: We will use the information that $T[0] = 0$ and parabolic symmetry to find an alternate expression for $T[1]$ to substitute into the equation for $T[2]$ therefore allowing $T[2]$ to act as a proxy boundary. From the master equation, we have-

$$T[1] = \tau + r_s'(T[2]) + r_h' \sum_{x=3}^{L-1} T[x]$$

Where r_s' and r_h' are the values of r_s and r_h when $x \in \{1, L-1\}$. We will use the symmetry property to write $T[L-1] = T[1]$, we then add and subtract $r_h'(T[2])$ to transform the above equation into the form

$$(1 - r_h')T[1] = \tau + (r_s' - r_h')T[2] + r_h' \sum_{x=2}^{L-2} T[x]$$

The summation term can be evaluated as follows

$$\sum_{x=2}^{L-2} T[x] = \sum_{x=2}^{L-2}\left[\frac{\beta}{\alpha} + C_1(e^{x\sqrt{\alpha}} + e^{(L-x)\sqrt{\alpha}})\right]$$
$$= (L-3)\frac{\beta}{\alpha} + C_1(e^{2\sqrt{\alpha}} + e^{3\sqrt{\alpha}} + ... + e^{(L-3)\sqrt{\alpha}} + e^{(L-2)\sqrt{\alpha}} +$$
$$e^{(L-2)\sqrt{\alpha}} + e^{(L-3)\sqrt{\alpha}} + ... + e^{3\sqrt{\alpha}} + e^{2\sqrt{\alpha}})$$
$$= (L-3)\frac{\beta}{\alpha} + 2C_1(e^{2\sqrt{\alpha}} + e^{3\sqrt{\alpha}} + ... + e^{(L-3)\sqrt{\alpha}} + e^{(L-2)\sqrt{\alpha}})$$

Using geometric progression series summation-

$$\sum_{x=2}^{L-2} T[x] = (L-3)\frac{\beta}{\alpha} + 2C_1\left[e^{2\sqrt{\alpha}}\left(\frac{e^{(L-3)\sqrt{\alpha}}-1}{e^{\sqrt{\alpha}}-1}\right)\right]$$

We will denote-

$$\psi = \left[e^{2\sqrt{\alpha}}\left(\frac{e^{(L-3)\sqrt{\alpha}}-1}{e^{\sqrt{\alpha}}-1}\right)\right]$$

and rewrite the equation as-

$$\sum_{x=2}^{L-2} T[x] = (L-3)\frac{\beta}{\alpha} + 2C_1\psi \tag{S6}$$

Using Eq. (S6) the summation in $T[1]$ can be rewritten as

$$T[1] = \frac{1}{(1-r'_h)}\left[\tau + (r'_s - r'_h)T[2] + r'_h(L-3)\frac{\beta}{\alpha} + 2r'_hC_1\psi\right]$$

but $r'_h = \frac{p}{2+p(L-3)}$ and $r'_s = \frac{1}{2+p(L-3)}$, therefore

$$\frac{1}{1-r'_h} = \frac{1}{1-\frac{p_u}{2+p_u(L-3)}} = \frac{2+p_u(L-3)}{2+p_u(L-4)} = \frac{2+p_u(L-4)+p_u}{2+p_u(L-4)} = 1 + r_h$$

similarly we can derive

$$\frac{r'_s - r'_h}{1-r'_h} = r_s - r_h, \qquad \frac{r'_h}{1-r'_h} = r_h$$

Hence we can rephrase $T[1]$ as

$$T[1] = (1+r_h)\tau + (r_s - r_h)T[2] + r_h(L-3)\frac{\beta}{\alpha} + 2r_hC_1\psi \tag{S7}$$

We will be using the master equation of $T[2]$ to solve for C_1

$$T[2] = \tau + r_sT[1] + r_sT[3] + s\,(T[4] + T[5] + ... + T[L-2]) + r_hT[L-1]$$

To this equation we will add and subtract $r_h(T[2]+T[3])$ to obtain a summation term that we have already evaluated, we then use the property that $T[1] = T[L-1]$ to get

$$(1+s)T[2] = \tau + (r_s + r_h)T[1] + (r_s - r_h)T[3] + r_h\sum_{x=2}^{L-2} T[x] \quad \text{(Make a substitution using Eq. (S6))}$$
$$= \tau + (r_s + r_h)T[1] + (r_s - r_h)T[3] + r_h(L-3)\frac{\beta}{\alpha} + 2r_hC_1\psi$$

Making the appropriate substitution for $T[1]$ from Eq. (S7) results in

$$(1+r_h)T[2] = \tau + (r_s+r_h)\left((1+r_h)\tau + (r_s-r_h)T[2] + r_h(L-3)\frac{\beta}{\alpha} + 2r_hC_1\psi\right)$$
$$+ (r_s-r_h)T[3] + r_h(L-3)\frac{\beta}{\alpha} + 2r_hC_1\psi$$

This equation can be rearranged into the form

$$\left(1+r_h-r_s^2+r_h^2\right)T[2] = \tau\left(1+(r_s+r_h)(1+r_h)\right) + (1+r_s+r_h)r_h\left((l-3)\frac{\beta}{\alpha} + 2C_1\psi\right) + (r_s-r_h)T[3]$$

We will substitute the values of $T[2]$ and $T[3]$ using Eq. (S5) and bringing the C_1 terms to the left hand side gives

$$C_1\left(\left[1+r_h-r_s^2+r_h^2\right]\left(e^{2\sqrt{\alpha}} + e^{(L-2)\sqrt{\alpha}}\right) - 2\psi r_h(1+r_s+r_h) - (r_s-r_h)\left(e^{3\sqrt{\alpha}} + e^{(L-3)\sqrt{\alpha}}\right)\right) =$$
$$\tau\left(1+(r_s+r_h)(1+r_h)\right) + \frac{\beta}{\alpha}\left((1+r_s+r_h)r_h(L-3) + r_s^2 + r_s - (r_h+1)^2\right)$$

which implies that

$$C_1 = \frac{\tau\left(1+(r_s+r_h)(1+r_h)\right) + \frac{\beta}{\alpha}\left((1+r_s+r_h)r_h(L-3) + r_s^2 + r_s - (r_h+1)^2\right)}{\left[1+r_h-r_s^2+r_h^2\right]\left(e^{2\sqrt{\alpha}} + e^{(L-2)\sqrt{\alpha}}\right) - 2\psi r_h(1+r_s+r_h) - (r_s-r_h)\left(e^{3\sqrt{\alpha}} + e^{(L-3)\sqrt{\alpha}}\right)}$$

For ease in further calculations we define,

$$\phi_1 = 1 + (r_s+r_h)(1+r_h)$$

$$\phi_2 = (1+r_s+r_h)r_h(L-3) + r_s^2 + r_s - (r_h+1)^2$$

$$\phi_3 = \left[1+r_h-r_s^2+r_h^2\right]\left(e^{2\sqrt{\alpha}} + e^{(L-2)\sqrt{\alpha}}\right) - 2\psi r_h(1+r_s+r_h) - (r_s-r_h)\left(e^{3\sqrt{\alpha}} + e^{(L-3)\sqrt{\alpha}}\right)$$

and rewrite C_1 as

$$C_1 = \frac{\phi_1\tau + \frac{\phi_2\beta}{\alpha}}{\phi_3} \tag{S8}$$

Now we can evaluate for $\beta = \frac{\tau + r_h\sum_{x=1}^{L-1}T[x]}{r_s-r_h}$ as follows

$$(r_s-r_h)\beta = \tau + r_h\sum_{x=1}^{L-1}T[x]$$
$$(r_s-r_h)\beta = \tau + r_h\sum_{x=2}^{L-2}T[x] + r_h(T[1]+T[L-1])$$
$$(r_s-r_h)\beta = \tau + r_h\left((L-3)\frac{\beta}{\alpha} + 2C_1\psi\right) + 2r_hT[1]$$

substitute values of C_1, $T[1]$ and $T[2]$ to get

$$(r_s-r_h)\beta = \tau + r_h\left((L-3)\frac{\beta}{\alpha} + 2\left[\frac{\phi_1\tau + \frac{\phi_2\beta}{\alpha}}{\phi_3}\right]\psi\right)$$
$$+2r_h((1+r_h)\tau + (r_s-r_h)\left(\frac{\beta}{\alpha} + \left[\frac{\phi_1\tau + \frac{\phi_2\beta}{\alpha}}{\phi_3}\right](e^{2\sqrt{\alpha}} + e^{(L-2)\sqrt{\alpha}})\right) + r_h(L-3)\frac{\beta}{\alpha} + 2r_h\left[\frac{\phi_1\tau + \frac{\phi_2\beta}{\alpha}}{\phi_3}\right]\psi)$$

Taking β terms to LHS gives

$$\beta\left[r_s - r_h - \frac{r_h(L-3)(1+2r_h)}{\alpha} - \frac{2r_h\phi_2\psi(1+2r_h)}{\alpha\phi_3} - \frac{2r_h(r_s-r_h)}{\alpha}\left(1+\frac{\phi_2(e^{2\sqrt{\alpha}}+e^{(L-2)\sqrt{\alpha}})}{\phi_3}\right)\right] =$$
$$\tau\left(1+2r_h(1+r_h)+\frac{2r_h\phi_1\psi(1+2r_h)}{\phi_3}+2r_h(r_s-r_h)\frac{\phi_1(e^{2\sqrt{\alpha}}+e^{(L-2)\sqrt{\alpha}})}{\phi_3}\right)$$

Which implies β is given by the equation

$$\beta = \frac{\tau\left(1+2r_h(1+r_h)+\frac{2r_h\phi_1\psi(1+2r_h)}{\phi_3}+2r_h(r_s-r_h)\frac{\phi_1(e^{2\sqrt{\alpha}}+e^{(L-2)\sqrt{\alpha}})}{\phi_3}\right)}{r_s-r_h-\frac{r_h(L-3)(1+2r_h)}{\alpha}-\frac{2r_h\phi_2\psi(1+2r_h)}{\alpha\phi_3}-\frac{2r_h(r_s-r_h)}{\alpha}\left(1+\frac{\phi_2(e^{2\sqrt{\alpha}}+e^{(L-2)\sqrt{\alpha}})}{\phi_3}\right)}$$

With all the unknowns identified in terms of known variables, we can write the closed-form solution in the domain $p_u \in (0,1)$ as

$$T[x] = \frac{\beta}{\alpha} + \left(\frac{\phi_1\tau + \frac{\phi_2\beta}{\alpha}}{\phi_3}\right)(e^{x\sqrt{\alpha}} + e^{(L-x)\sqrt{\alpha}}) \tag{S9}$$

This solution is in the domain $x \in [2, L-2]$ but we can calculate values at $T[1]$ and $T[L-1]$ by using Eq.(S7).

S2.2.1 Small p_u approximation

The general solution of our differential equation is given by Eq. (S4),

$$T[x] = \frac{\beta}{\alpha} + C_1 e^{x\sqrt{\alpha}} + C_2 e^{-x\sqrt{\alpha}}$$

For $p_u << 1/L$ we can make the assumption that $r'_s = r_s$ and $r'_h = r_h$, this would help us remove the discontinuity observed at positions $i \in \{1, L-1\}$

Boundary condition 1: Same as in the previous case the first derivative of T at $x = \frac{L}{2}$ is zero

$$\Rightarrow \frac{\partial T[L/2]}{\partial x} = \sqrt{\alpha}\left(C_1 e^{\frac{\sqrt{\alpha}L}{2}} - C_2 e^{-\frac{\sqrt{\alpha}L}{2}}\right) = 0$$

from the above equation we can derive $\left[C_2 = C_1 e^{\sqrt{\alpha}L}\right]$ and substitute in Eq.(S4) to get

$$T[x] = \frac{\beta}{\alpha} + C_1(e^{x\sqrt{\alpha}} + e^{(L-x)\sqrt{\alpha}})$$

Boundary condition 2: We have defined $T[0] = 0$, using this condition

$$T[0] = \frac{\beta}{\alpha} + C_1(1 + e^{L\sqrt{\alpha}}) = 0$$
$$\Rightarrow C_1 = \frac{-\beta}{\alpha(1 + e^{L\sqrt{\alpha}})}$$

Now the only remaining unknown is β. We will evaluate this making use of the information

$$\beta = \frac{\tau + r_h \sum_{x=1}^{L-1} T[x]}{r_s - r_h} \tag{S10}$$

The summation term in Eq.(S10) can be evaluated as follows

$$\sum_{x=1}^{L-1} T[x] = \sum_{x=1}^{L-1}\left[\frac{\beta}{\alpha} + \frac{-\beta}{\alpha(1+e^{L\sqrt{\alpha}})}(e^{x\sqrt{\alpha}} + e^{(L-x)\sqrt{\alpha}})\right]$$
$$= \frac{\beta}{\alpha}[L-1-\frac{1}{1+e^{L\sqrt{\alpha}}}(e^{\sqrt{\alpha}} + e^{2\sqrt{\alpha}} + ... + e^{(L-2)\sqrt{\alpha}}$$
$$+ e^{(L-1)\sqrt{\alpha}} + e^{(L-1)\sqrt{\alpha}} + e^{(L-2)\sqrt{\alpha}} + ... + e^{2\sqrt{\alpha}} + e^{\sqrt{\alpha}})]$$
$$= \frac{\beta}{\alpha}\left[L-1-\frac{2(e^{\sqrt{\alpha}} + e^{2\sqrt{\alpha}} + ... + e^{(L-2)\sqrt{\alpha}} + e^{(L-1)\sqrt{\alpha}})}{1+e^{L\sqrt{\alpha}}}\right]$$

Using geometric progression series summation

$$\sum_{x=1}^{L-1} T[x] = \frac{\beta}{\alpha}\left[L-1-\frac{2e^{\sqrt{\alpha}}(e^{\sqrt{\alpha}(L-1)}-1)}{(1+e^{L\sqrt{\alpha}})(e^{\sqrt{\alpha}}-1)}\right]$$

We will denote

$$\rho = L-1-\frac{2e^{\sqrt{\alpha}}(e^{\sqrt{\alpha}(L-1)}-1)}{(1+e^{L\sqrt{\alpha}})(e^{\sqrt{\alpha}}-1)} \tag{S11}$$

and rewrite Eq. (S10) as

$$\sum_{x=1}^{L-1} T[x] = \frac{\beta}{\alpha}\rho \tag{S12}$$

Substituting Eq. (S12) in the equation for β (Eq. (S10)),

$$\beta = \frac{\tau + r_h\frac{\beta}{\alpha}\rho}{r_s - r_h}$$
$$\Rightarrow \beta = \frac{\tau}{r_s - r_h\left(1+\frac{\rho}{\alpha}\right)}$$

Hence, we show that when p_u takes values which are much less than $\frac{1}{L}$,

$$T[x] = \frac{\tau}{\alpha\left(r_s - r_h\left(1+\frac{\rho}{\alpha}\right)\right)}\left[1-\frac{e^{x\sqrt{\alpha}} + e^{(L-x)\sqrt{\alpha}}}{1+e^{L\sqrt{\alpha}}}\right]$$
$$= \frac{\tau}{\alpha(r_s - r_h) - r_h\rho}\left[1-\frac{e^{x\sqrt{\alpha}} + e^{(L-x)\sqrt{\alpha}}}{1+e^{L\sqrt{\alpha}}}\right] \tag{S13}$$

where ρ is given by Eq. (S11).

S2.2.2 Large p_u approximation

For larger p_u values we can make a simple approximation that $r_s \approx r_h$ and $T[2] = T[3] = ... = T[L-2]$. Applying these to Eq.(S2) gives

$$-(1+3r_h-2r_s)\,T[x] + (\tau + r_h(L-3)T[x] + 2r_h(T[1])) = 0 \tag{S14}$$

But from the master equation

$$T[1] = \tau + r_s' T[2] + r_h'(T[3] + T[4] + ... + T[L-1])$$

Using the same approximations $r_s \approx r_h$ and $T[2] = T[3] = ... = T[L-2]$ we can evaluate the above expression to get

$$T[1] = \frac{\tau + (r'_s + (L-4)r'_h)T[x]}{1 - r'_h}$$

Substituting in Eq. (S14) and solving for T[x] yields

$$T[x] = \frac{(1 + \frac{2r_h}{1-r'_h})\tau}{1 - 2r_s - r_h(L-6) - \frac{2r_h(r'_s+(L-4)r'_h)}{1-r'_h}}$$

Assuming $\tau = 1$ the above equation can be simplified by using the definition of r_s, r'_s, r_h and r'_h in terms of L and p_u be rewritten as

$$T[x] = 2 + (L-3)p_u + \frac{(2 + (L-4)p_u)^2}{2p_u} \tag{S15}$$

S2.2.3 The effective medium approach at large p_u

The Erdős–Rényi (ER) network is a mathematical model of random graphs in graph theory. In this model, each pair of nodes in a graph is connected with a certain probability, and the resulting graph is a random realization of this probability. In the high-p_u limit, we take the nodes $i \in [1, L-1]$ of polymer domain to be an Erdős–Rényi network with $L-1$ nodes and calculate mean time to reach boundary nodes, following the approach described in [4].

Let τ_{ab} be the mean transit time to reach any arbitrary node b starting from a by a random walker in this Erdős–Rényi network with $L-1$ nodes having connection probability p_u under the case where a and b is directly connected by a path. The number of paths coming out of a excluding the adjacent nodes are $(L-4)p_u$, the adjacent nodes are always connected. The probability to reach b starting from a in a single step using the direct connection is $\frac{1}{2+(L-4)p_u}$. On the other hand, if the walker avoids the direct path between a and b, the time is given by $\left(1 - \frac{1}{2+(L-4)p_u}\right)\tau'_{ab}$. Here τ'_{ab} is the average time to reach b from a in absence of direct path. Then we have [4],

$$\tau'_{ab} = p_u(\tau_{ab} + 1) + (1 - p_u)(\tau'_{ab} + 1) \tag{S16}$$

This allows to write a recursion relation of τ_{ab}

$$\tau_{ab} = \frac{1}{2 + (L-4)p_u} + \left(1 - \frac{1}{2 + (L-4)p_u}\right)\left[p_u(\tau_{ab} + 1) + (1 - p_u)(\tau'_{ab} + 1)\right] \tag{S17}$$

Solving these two equations Eq. (S16) and Eq. (S17) we get

$$\tau_{ab} = L - 3 + \frac{1}{p_u} \qquad \tau'_{ab} = L - 3 + \frac{2}{p_u}$$

Hence, the average time taken by the walker to reach b starting from a in a Erdős–Rényi network is

$$\langle T_{ab} \rangle_{\mathrm{ER}} = p_u\tau_{ab} + (1 - p_u)\tau'_{ab} = L - 4 + \frac{2}{p_u} \tag{S18}$$

We are only concerned about the time it takes to reach either one of our near boundary nodes, that is 1 or L, assuming b to be either one of these we can argue that

$$\langle T \rangle_{\mathrm{ER}} = \frac{(L-4)p_u + 2}{2p_u} \tag{S19}$$

We define $\langle T \rangle$ to be the mean time taken to make an exit from the chain. In this case, $\langle T \rangle$ follows the following relation–

$$\langle T \rangle = \frac{1}{2 + (L-3)p_u} + \left(1 - \frac{1}{2 + (L-3)p_u}\right) [1 + \langle T \rangle_{\mathrm{ER}} + \langle T \rangle] \tag{S20}$$

Substituting $\langle T \rangle_{\mathrm{ER}}$ and solving for $\langle T \rangle$ gives us

$$\langle T \rangle = 1 + \frac{(1 + (L-3)p_u)(2 + (L-2)p_u)}{2p_u} \tag{S21}$$

This estimate of mean search time matches well with the simulation for the higher p_u ($p_u \to 1$) (S5 Fig). However for smaller p_u values, the effective medium approach breaks down due to decreased connectivity of the network.

S2.3 The effect of 3D unbinding on mean search time

The general facilitated diffusion model proposes bulk diffusion as one of the major modes of protein search. However, experiments have shown that the percentage of the time that the protein spends bound to DNA is greater than 70%. For example, RNA polymerase has been reported to remain bound to DNA for approximately 87.2–93% of the time [5, 1]. Similarly, the Lac repressor exhibits DNA-bound fractions in the range of 87–96% [5, 2]. p53 displays a broader range of DNA occupancy, with reported bound fractions spanning from 72% to nearly 99.9%, depending on the experimental conditions [3, 6]. This is very different from the most optimal conditions predicted by the facilitated diffusion model.

General facilitated diffusion models do not consider the polymer organisation of chromatin. In order to compare the effects of both 3D diffusion and polymer compaction in regulating search times, we calculate the mean search time in the presence of both of these effects. For dynamic polymer configurations, the mean search time can be described the recursion relation,

$$\mathbb{T}_i = \tau + \frac{1 - p_{\mathrm{off}}}{N_i} \left[\mathbb{T}_{i-1} + \mathbb{T}_{i+1} + \sum_{j \neq nn, 0, L} c|i-j|^{-\gamma} \mathbb{T}_j \right] + p_{\mathrm{off}} \mathbb{T}_f \tag{S22}$$

When the walker is at position i the dynamic average of possible connections

$$N_i = 2 + \sum_{j \neq nn, 0, L} c|i-j|^{-\gamma} \quad \forall\ i, j \in [1, L-1]$$

and $\mathbb{T}_f$ is the search time with polymer starting in the bulk and is defined as,

$$\mathbb{T}_f = \tau_f + \frac{\sum_{j=0}^{L} \mathbb{T}_j}{L+1}$$

By introducing $p_{\mathrm{off}} \neq 0$, we allow protein to go to solution with unbinding probability p_{off}. Upon unbinding, the protein rebinds randomly to any bead on chromatin after a characteristic time τ_f. We assume $\tau_f = 100\tau$ [7], although our results do not depend on the specific choice of τ_f.

References

[1] Kelsey Bettridge et al. "RNAP promoter search and transcription kinetics in live E. coli cells". In: *The Journal of Physical Chemistry B* 127.17 (2023), pp. 3816–3828.

[2] Johan Elf, Gene-Wei Li, and X Sunney Xie. "Probing transcription factor dynamics at the single-molecule level in a living cell". In: *Science* 316.5828 (2007), pp. 1191–1194.

[3] Kiyoto Kamagata et al. “Single-molecule characterization of target search dynamics of DNA-binding proteins in DNA-condensed droplets”. In: *Nucleic Acids Research* 51.13 (2023), pp. 6654–6667.

[4] V Sood, S Redner, and D ben-Avraham. “First-passage properties of the Erdős–Renyi random graph”. In: *Journal of Physics A: Mathematical and General* 38.1 (Dec. 2004), p. 109. DOI: 10.1088/0305-4470/38/1/007. URL: https://dx.doi.org/10.1088/0305-4470/38/1/007.

[5] Mathew Stracy et al. “Transient non-specific DNA binding dominates the target search of bacterial DNA-binding proteins”. In: *Molecular cell* 81.7 (2021), pp. 1499–1514.

[6] Anahita Tafvizi et al. “A single-molecule characterization of p53 search on DNA”. In: *Proceedings of the National Academy of Sciences* 108.2 (2011), pp. 563–568.

[7] Alex Veksler and Anatoly B Kolomeisky. “Speed-selectivity paradox in the protein search for targets on DNA: is it real or not?” In: *The journal of physical chemistry B* 117.42 (2013), pp. 12695–12701.
