# Supplementary figures and images for: Compaction of chromatin domains regulates target search times of proteins
Source: PLoS Comput Biol. 2026 Jan 20;22(1):e1013843. doi: 10.1371/journal.pcbi.1013843 (PMC12858080; doi:10.1371/journal.pcbi.1013843)

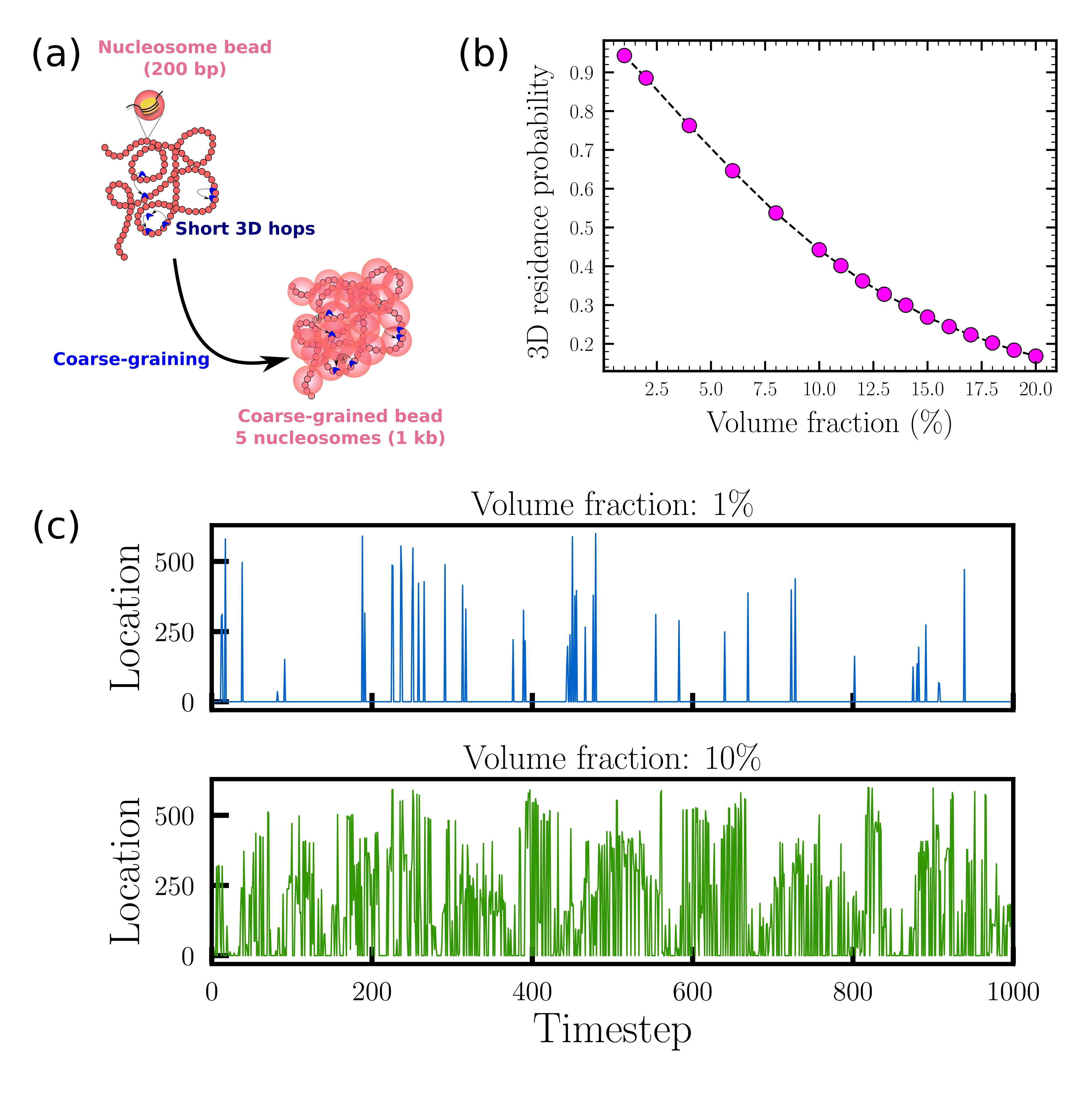

Supplement: S1 Fig — (a) Schematic of the construction of a coarse-grained chromatin polymer, where each bead represents 1 kb, obtained from fine-grained nucleosome beads (200 bp). Short-range hopping events appear as intersegmental jumps in the coarse-grained representation. (b) Residence probability in 3D diffusion mode, where the protein is not bound to chromatin, varies as a function of the volume fraction of chromatin in the box. As the volume fraction increases, bulk exploration decreases due to the limited space for free movement in densely packed chromatin regions. (c) Representative protein trajectories illustrating the motion at volume fractions of 1% and 10%. At low volume fractions, motion is primarily governed by binding and unbinding processes. However, at high volume fractions, the motion exhibits numerous intersegmental jumps. (TIFF) [file pcbi.1013843.s003.tif]

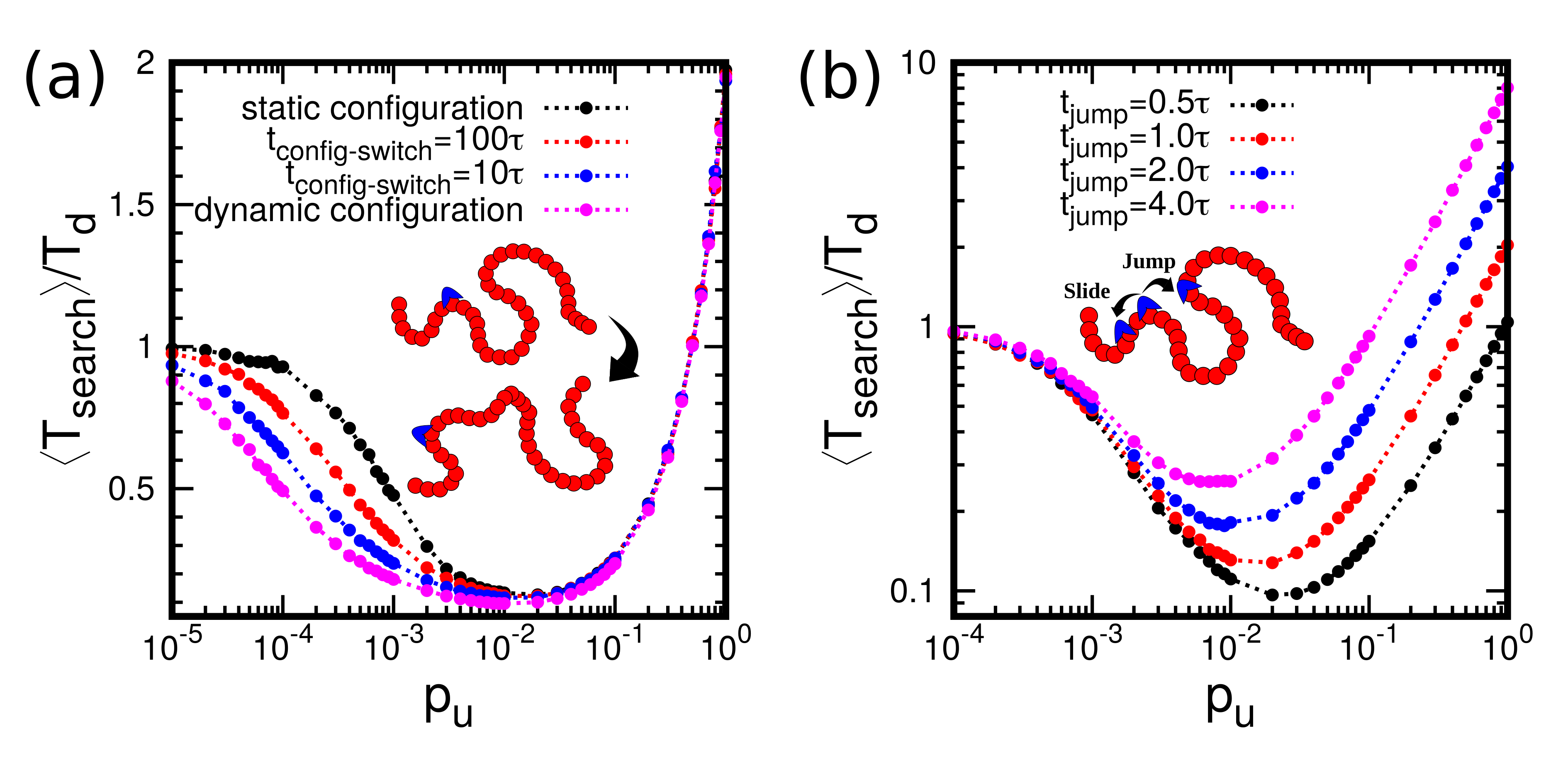

Supplement: S2 Fig — (a) ⟨Tsearch⟩Td in uniformly connected network domain vs pu for different dynamic rewiring for L = 100. The non-monotonic behaviour of ⟨Tsearch⟩ remains robust even when the polymer network undergoes dynamic re-configurations at a fixed connection probability. Dynamic rewiring of network connections reduces ⟨Tsearch⟩, with faster configuration changes yielding lower ⟨Tsearch⟩ at low pu values. However, at high pu values, network dynamicity offers little advantage, as most nodes already have numerous long-range connections. (b) ⟨Tsearch⟩Td in uniformly connected network domain vs pu for different jump times for L = 100. We show that the non-monotonic behaviour of ⟨Tsearch⟩ persists even when sliding along the backbone and jumps along intersegmental bonds have different timescales. At low pu, the ⟨Tsearch⟩ remains independent of tjump, dominated by 1D sliding. Conversely, for higher pu, where jumps are more probable than sliding, ⟨Tsearch⟩ increases as the tjump increases. (TIFF) [file pcbi.1013843.s004.tif]
